# Potent anticancer activity of a novel iridium metallodrug via oncosis

**DOI:** 10.1007/s00018-022-04526-5

**Published:** 2022-09-06

**Authors:** Enrique Ortega-Forte, Samanta Hernández-García, Gloria Vigueras, Paula Henarejos-Escudero, Natalia Cutillas, José Ruiz, Fernando Gandía-Herrero

**Affiliations:** 1 Departamento de Química Inorgánica, Universidad de Murcia, and Murcia BioHealth Research Institute (IMIB-Arrixaca), 30071 Murcia, Spain; 2 Departamento de Bioquímica y Biología Molecular A. Unidad Docente de Biología, Facultad de Veterinaria, Universidad de Murcia, 30071 Murcia, Spain

**Keywords:** Iridium metallodrug, Oncosis, Anticancer agents, Antitumor activity, *Caenorhabditis elegans*

## Abstract

Oncosis (from Greek *ónkos*, meaning “swelling”) is a non-apoptotic cell death process related to energy depletion. In contrast to apoptosis, which is the main form of cell death induced by anticancer drugs, oncosis has been relatively less explored but holds potential to overcome drug resistance phenomena. In this study, we report a novel rationally designed mitochondria-targeted iridium(III) complex (**OncoIr3**) with advantageous properties as a bioimaging agent. **OncoIr3** exhibited potent anticancer activity in vitro against cancer cells and displayed low toxicity to normal dividing cells. Flow cytometry and fluorescence-based assays confirmed an apoptosis-independent mechanism involving energy depletion, mitochondrial dysfunction and cellular swelling that matched with the oncotic process. Furthermore, a *Caenorhabditis elegans* tumoral model was developed to test this compound in vivo, which allowed us to prove a strong oncosis-derived antitumor activity in animals (with a 41% reduction of tumor area). Indeed, **OncoIr3** was non-toxic to the nematodes and extended their mean lifespan by 18%. Altogether, these findings might shed new light on the development of anticancer metallodrugs with non-conventional modes of action such as oncosis, which could be of particular interest for the treatment of apoptosis-resistant cancers.

**Graphical abstract:**

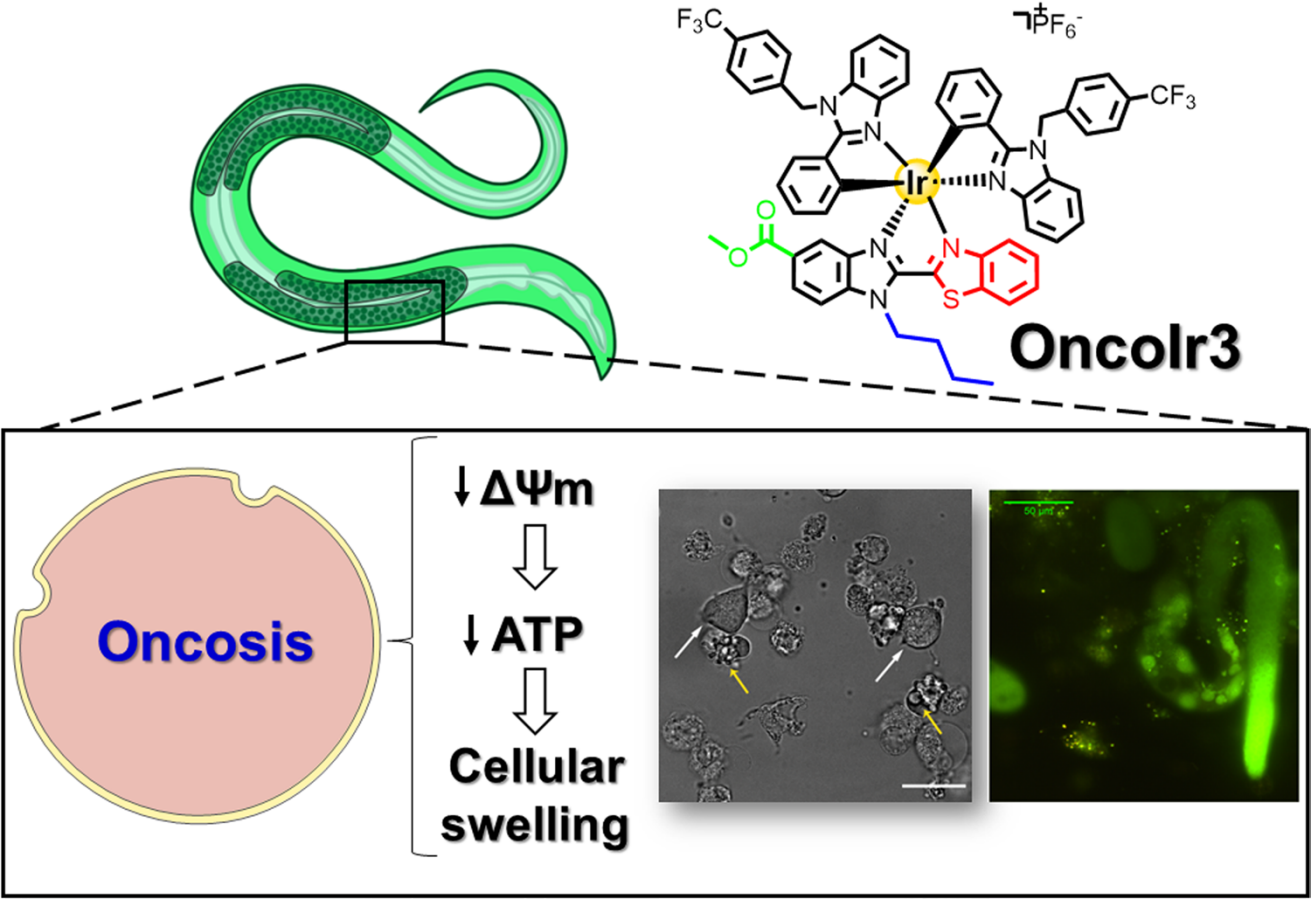

**Supplementary Information:**

The online version contains supplementary material available at 10.1007/s00018-022-04526-5.

## Introduction

Cancer cells evolve multiple mechanisms to evade apoptosis [1]. This is one of the main factors leading to drug resistance, because most clinical anticancer chemotherapeutics act via apoptosis induction [2]. Consequently, there is an urgent need for new drug candidates that induce cancer cell death through non-apoptotic mechanisms. In this sense, metallodrugs have gained increased attention as potential anticancer agents since they can exhibit a variety of alternative modes of action, including translation inhibition [3], ferroptosis [4], necroptosis [5] or paraptosis [6].

In 2018, a family of cyclometalated iridium(III) compounds bearing benzothiazole ligands were reported for the first time as inducers of a novel, non-apoptotic type of cell death known as oncosis [7]. Recently, an osmium(IV) nitride compound was also found to elicit oncosis in cancer cells [8]. Oncosis (from Greek *ónkos*, meaning “swelling”) refers to a cell death process related to energy depletion [9, 10]. This cell death mode is characterized by mitochondrial dysfunction and reduction of adenosine triphosphate (ATP) levels, which results in loss of ionic homeostasis, increased membrane permeability and eventual cellular swelling [9]. Oncosis is emerging as a novel approach to kill cancer cells and has demonstrated to be effective for resistant cancers [11, 12, 13].

**Fig. 1:**
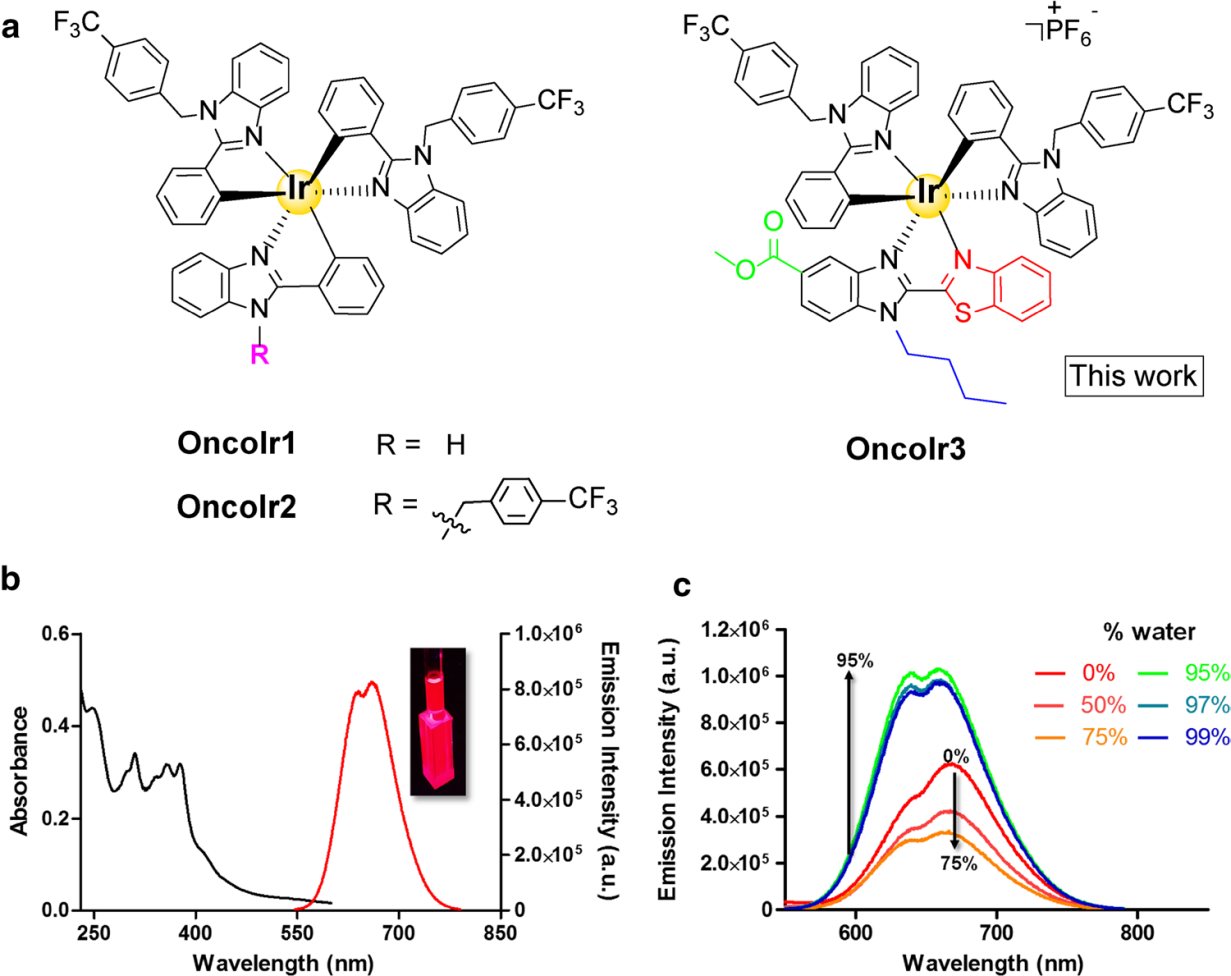
Chemical structure and photophysical characterization of **OncoIr3**. **a** The reported oncosis-like neutral inducers **OncoIr1**, **OncoIr2** and our current work **OncoIr3**. **b** Absorption and emission spectra of **OncoIr3** in water (1% DMSO). (**c**) Emission spectra of **OncoIr3** in DMSO/water mixtures with different water fractions (fw). *λ*_exc_ = 405 nm

Very recently, some of us reported two new iridium compounds, namely **OncoIr1** and **OncoIr2** (Fig. 1a), which also induced oncosis-like cell death [14]. However, these two compounds were unable to enter human cells, and only displayed their anticancer activity upon nanoparticle encapsulation [14]. Based on these results, we decided to redesign the chemical structure of **OncoIr1** and **OncoIr2** to improve cellular uptake into cancer cells and induce cell death via oncosis. In this study, we report a novel luminescent iridium(III) complex (**OncoIr3**) incorporating a benzothiazole ligand which exhibits potent anticancer activity in vitro. Furthermore, a *Caenorhabditis elegans* tumoral model was developed to test this compound in vivo, which allowed us to confirm a strong oncosis-derived antitumor activity in animals. Altogether, these findings might shed new light on the development of anticancer metallodrugs with non-conventional modes of action such as oncosis, which could be of particular interest for the treatment of apoptosis-resistant cancers.

## Materials and methods

### Cell lines and culture

Human ovarian carcinoma cell lines, A2780 and A2780cis, were grown in RPMI-1640 medium. Human cervix adenocarcinoma cell line, HeLa, tumor breast cancer cell line, MDA-MB-231, and non-tumorigenic buffalo green monkey cells, BGM, were grown in DMEM medium. Chinese hamster ovary cells, CHO, were grown in FK-12 medium. All the cell media were supplemented with 10% fetal bovine serum (FBS), 1% l-glutamine and 1% penicillin/streptomycin. The acquired resistance of A2780cis cells was maintained by supplementing the culture medium with 1 μM cisplatin every second passage. The cell lines were cultured in a humidified incubator at 37ºC in a 5% CO_2_ atmosphere and subcultured 2–3 times a week with an appropriate density for each cell line. The cell lines were confirmed to be mycoplasma-free using Hoechst DNA staining standard methodologies. In all cell-based assays, the amount of DMSO added as treatment solvent was kept at a maximum of 0.4% (v/v) to avoid vehicle toxicity to the cells.

### In vitro antiproliferative activity

Cell viability was determined using a thiazolyl-blue tetrazolium bromide (MTT)-reagent to assess cell vitality upon exposure to the tested compounds. Cells were cultured in 96-well plates at a density of 5 × 10^3^ cells/well in complete medium and incubated for 24 h. Serial dilutions of chemical compounds were prepared using DMSO (**OncoIr3**) or water (cisplatin) as a solvent. These dilutions were further diluted in cell media and added at the final concentrations in the range of 0–100 μM in a final volume of 100 μL per well and incubated for 48 h. Cell media was then removed and 50 µL MTT (1 mg/mL) was added to each well. After 4 h incubation, the MTT solution was removed and 50 µL DMSO was added to solubilize the purple formazan crystals formed in active cells. The absorbance was measured at 570 nm using a microplate reader (FLUOstar Omega) and the IC_50_ values were calculated based on the inhibitory rate curves using the equation:

$$I = \frac{{I_{\max } }}{{1 + \left( {\frac{{IC_{50} }}{C}} \right)^{n} }},$$

where *I* represents the percentage inhibition of viability observed, *I*_max_ is the maximal inhibitory effect, *IC*_*50*_ is the concentration that inhibits 50% of maximal growth, *C* is the concentration of the compound and *n* is the slope of the semi-logarithmic dose–response sigmoidal curves. The non-linear fitting was performed using SigmaPlot 14.0 software. Three independent experiments with each cell line for either **OncoIr3** and cisplatin were run within a week of each other with n = 3 replicates per concentration level.

### Fluorescence imaging

All fluorescence microscopy observations were performed using a STELLARIS 8 Leica Microsystems confocal microscope equipped with a 405 nm laser diode, an argon-ion laser, and a 488 nm laser. The microscope was also equipped with temperature and CO_2_ providing system. A2780 and HeLa cells were seeded onto ibidi-plates and allowed to reach confluence. Cells were then imaged at 37 ºC using a 63 × glycerol immersion objective. In colocalization studies, Mitotracker Green FM staining (0.1 µM in PBS; 30 min) was observed with the 488 nm laser line, whereas the 405 nm laser diode was used for **OncoIr3** detection. Colocalization coefficients were calculated using the JaCoP plugin in Fiji software on the different stacks of images (*n* = 14) with each stack containing at least 5 cells.

### Metal content determination in cancer cells

A2780 cells were seeded in T25 cm^2^ flasks at high density and allowed to reach 80% confluence over 48 h. Cells were then treated with 5 or 10 µM of the tested compound and incubated at 4 ºC or 37 ºC for 2 h. Cells were trypsinized, counted and the samples were digested using Suprapur® nitric acid 30% for 2 h. The amount of metal element iridium was determined in triplicate using Inductively Coupled Plasma Mass Spectrometry (ICP-MS) Agilent 7900 series.

### Cell morphology examination

To evaluate cell morphology, A2780 cancer cells were seeded in 6-well plates at 2.5 × 10^5^cells/well and incubated overnight. The following day, cells were treated with either the complex or cisplatin and morphological changes were assessed after 24 h, when cells were collected by trypsinization and subjected to flow cytometry (Beckman CoulterEpics XL; 10^4^ events per sample). Acquisitions were recorded and analyzed by plotting both forward and light scatter (FSC vs SSC) in FlowingSoftware version 2.5.1. Three independent experiments were performed (*n* = 2). Alternatively, cells were imaged under phase-contrast microscopy using Axio inverted microscope.

### Mitochondrial membrane potential assays

Mitochondrial membrane potential was evaluated with the fluorescent probe JC-1 chloride (Promocell). A2780 cells (2 × 10^5^ cells/well) were seeded for 24 h in complete medium on 12-well plates, and then treated with various concentrations of the iridium complex or cisplatin for 24 h. Antimycin A (20 µM) was used as a positive control for mitochondrial dysfunction. After drug exposure, the cells were incubated with JC-1 dye (1 µM) for 20 min and subjected to flow cytometry (Becton Dickinson FACSCalibur; 10^4^ events acquired per sample), using *λ*_exc/em_ = 488/530 nm parameters to discriminate green JC1 monomers (FL1-H channel) and red JC1 aggregates (FL2-H channel). Three independent experiments were performed (*n* = 2).

### ATP determination

Intracellular ATP levels were determined using recombinant firefly luciferase bioluminescence assay according to manufacturer’s instructions (Invitrogen™). Briefly, A2780 cells (2 × 10^5^ cells/well) were seeded for 24 h in complete medium on 12-well plates, and then treated with various concentrations of the iridium complex or cisplatin for 24 h. Cells were then trypsinized and the pellets digested in chilled cell lysis buffer (eBiosience™). Intracellular content was then mixed with buffered standard solution reaction containing D-luciferin and luciferase as instructed. Luminescence at 560 nm was read in white 96 well plates using a microplate reader (FLUOstar Omega). Experiments were performed in triplicate (*n* = 3).

### Membrane permeability tests

A2780 cells were seeded in 12-well plates at a density of 2·10^5^ cells per well. After overnight incubation, cells were incubated with treatments at indicated concentrations and time. Then, cells were harvested by trypsinization, and the pellets were resuspended in propidium iodide solution (20 μg/mL) for 15 min. Samples were then subjected to flow cytometry (Beckton Dickinson FACSCalibur) with 10^4^ acquisitions in the FL2-H channel. Data were analyzed using FlowingSoftware version 2.5.1. The assay was performed in three independent experiments (*n* = 2 replicates).

### Cell cycle distribution analysis

A2780 cells (2.5 × 10^5^ cells/well) were seeded onto 12-well plates and allowed to attach overnight. Treatments at indicated concentrations were added for 24 h. Then cells were trypsinized and fixed in ice-cold ethanol 70% in PBS for 2 h. After centrifugation, a staining solution with 40 µg/mL Propidium Iodide and 1 µg/mL RNase were added for 30 min and the samples were subjected to analysis using FACS Calibur cytometer (BD Biosciences) with 10^4^ acquisitions per sample (*λ*_exc_ = 488 nm and *λ*_em_ = 630 nm). The analysis was performed using data from three independent experiences (*n* = 3).

### Cell death detection studies

Cell death induction was evaluated using the Annexin V/Propidium Iodide (AV/PI) labelling method. Briefly, A2780 cells were seeded in 12-well plates at a density of 2.5·10^5^ cells/well and incubated overnight. Iridium complex was added at indicated concentrations for 24 h and cisplatin was used as a positive control for apoptosis induction. After treatment, cells were harvested by trypsinization, washed with PBS, centrifuged and the pellets were resuspended in 180 µL binding buffer. Then, 10 µL Annexin-V-FITC (Promocell) and 10 µL propidium iodide were added and the resuspended cell solution was left at room temperature in the dark for 15 min. Cells were analyzed by flow cytometry using FACS Calibur cytometer (BD Biosciences); 10^4^ events acquired per sample, registering at 525 and 620 nm for AV and PI, respectively (*λ*_exc_ = 488 nm). Data were analyzed using FlowingSoftware version 2.5.1. Three independent experiments were performed (*n* = 3).

### Cell viability after co-incubation with chemical inhibitors

Cell viability assays were performed by the MTT method. A2780 cells seeded onto 96-well plates were incubated with different inhibitors, i.e., caspase inhibitor (NSCI, 5 μM), p53 inhibitor (pifithrin-alpha, 5 μM), translation inhibitor (cycloheximide, 50 μM), lysosomal protease inhibitor (leupeptin, 100 μM) or necroptosis inhibitor (Necrostatin-1, 60 μM) for 1 h. Following pretreatment, cell media were replaced by fresh media containing **OncoIr3** at indicated concentrations. After 24 h, cells were loaded with MTT solution for 4 h. DMSO was then used to solubilize formazan crystals and cell viability was determined based on 570 nm absorbance readings from three independent assays.

### Caenorhabditis elegans strains and maintenance

All the strains used in this work were obtained from the Caenorhabditis Genetic Center (CGC, St Paul, MN, USA), which is supported by the National Institutes of Health—Office of Research Infrastructure Programs (P40 OD010440). Wild-type N2, JK1466 (*gld-1(q485)/dpy-5(e61) unc-13(e51)*), CF1553 (*muIs84 [(pAD76) sod-3p::GFP* + *rol-6(su1006)]*) and TJ375 (*gpIs1 [hsp-16.2p::GFP]*) strains were used. The strains were maintained and synchronized following the published methods [15, 16]. *Escherichia coli* OP50 concentrated 10 × in M9 buffer was used to feed all the strains except the JK1466. The JK1466 strain was fed with the *E. coli* strain HT115 (DE3) with the homologous DNA sequence for the *gld-1* (T23G11.3) gene inside the vector L4440 (pPD129.36) was obtained from Source BioScience (sourcebioscience.com) from the library “RNAi Library (Ahringer)” to assure that all the worms had the tumoral phenotype following the published method [17].

### *C. elegans* assimilation of OncoIr3

The luminescent properties of **OncoIr3** allowed us to track assimilation by the animals. Age synchronized L4 larvae of wild-type worms were fed with *E. coli* OP50 and different concentrations of the compound (0.1, 1, 10 and 100 µM) in S basal medium for 48 h at 20 ºC. Then, worms were washed with M9 buffer three times and transferred to microscope slides containing sodium azide (10 mM) as anesthetic for its visualization. Fluorescent images of the animals were taken using the A filter cube of a Leica DM 2500 LED microscope fitted with a Leica DFC550 camera (Leica Microsystems, Wetzlar, Germany) with the 10 × lens. The quantification of the compound inside the worms was performed using ImageJ software [18]. The images were split in RGB channels and only the red channel was used to measure the fluorescence of the compound inside the worms using the threshold tool of the software. Two independent assays were performed with n ≥ 10 and the statistical significance was estimated by ANOVA test.

### Antitumoral evaluation in *C. elegans*

The evaluation of the antitumoral potential of the compound was performed as described [17]. Cisplatin was used as positive control. Briefly, JK1466 age synchronized L1 larvae was treated with the **OncoIr3** at different concentrations (0.1, 1, 10 and 100 µM) in S basal medium supplemented with previously induced *E. coli* HT115 *gld-1* at 20 ºC under orbital shaking. DMSO (0.4%) and water were used as negative controls and cisplatin (100 µM) as positive control. At the four day of adulthood, worms were washed in M9 buffer and visualized under the bright-field microscope. Images of the tumoral gonads were taken at 40 × and the size of the tumor was evaluated using the ImageJ software. Two independent assays were performed with *n* ≥ 10 and the statistical significance was estimated by ANOVA test.

Alternatively, *C. elegans* lifespan, both the tumoral strain and the wild-type strain was measured automatically using the lifespan machine [19] following the modifications of Guerrero-Rubio and coauthors [16]. Age-synchronized L4 larvae (30–40 individuals) were transferred to analysis plates containing NGM supplemented with 30 μg/mL of nystatin, 30 μg/mL of carbenicillin, FUdR (2′-deoxy-5-fluorouridine) at 10 μg/mL and IPTG was added to a final concentration of 1 mM to induce *E. coli* HT115 *gld-1* bacterial cells in JK1466 strain worms. Different concentrations of the compound (0.1, 1, 10 and 100 µM) were added for the test plates. DMSO (0.4%) was added to control plates. The plates for the wild-type assay were seeded with heat inactivated *E. coli* OP50 and the plates used for the JK1466 strain contained IPTG (1 mM) and were seeded with HT115 *gld-1*. At least two independent assays were performed, mathematical analysis of the obtained data was performed using the on-line application for survival analysis OASIS 2 [20], with the parameters Kaplan–Meier estimator and Survival Time F-Test.

### Oncosis detection in vivo

Morphology of *C. elegans* gonad cells was visualized using in vivo acridine orange staining [21]. Adult animals (4 days) were treated with the compound or cisplatin, washed in M9 buffer and transferred to 5 mL of fresh M9 containing 100 µL of concentrated *E. coli* OP50 and 5 µg/mL of acridine orange and left to stain for 1 h at 20 ºC under orbital shaking. After one hour the animals were washed with M9 three times and left in 5 mL of clean M9 for another hour to reduce the stain accumulated in the digestive tube. At least two independent experiments were performed with n ≥ 10.

## Results

### Design, synthesis and characterization of OncoIr3*.*

Based on the results obtained with **OncoIr1** and **OncoIr2** [14], we maintained two of the cyclometalating ligands fixed and modified the benzimidazole core of the third ligand for the molecular construction of the new iridium compound **OncoIr3** (Fig. 1a). First, the third ligand was replaced by an N^N ligand to change from neutral to a positively charged complex. The prospect of using a cationic cyclometalated iridium(III) compound is desirable since electronic-neutral molecules generally exhibit low water solubility, which usually hampers cell uptake [14, 22]. On the other hand, our previous results with bis-cyclometalated iridium(III) compounds demonstrated that slight modifications on benzimidazole-based C^N ligand core rendered high anticancer activities in vitro [23]. Iridium(III) complexes containing benzothiazole substituted ligands have also shown remarkable anticancer behaviour, particularly inducing oncosis [7]. Therefore, **OncoIr3** was designed to incorporate a N^N ligand with a benzothiazole group in the position 2 of benzimidazole (Fig. 1a). This ligand incorporation allowed to achieve an emission shift to the near-infrared region (~ 650–900 nm), which indeed enhances its operability as a bioimaging tool for in vitro and in vivo visualization. Previously reported modifications on benzimidazole-based metallodrugs demonstrated that an N-substituted butyl group can serve to adjust the lipophilic properties and enhance cellular uptake [23]. Finally, an ester group was attached to the benzimidazole backbone of **OncoIr3** to act as a handle for further functionalization.

The selected N^N benzothiazole ligand was synthesized by condensation of the intermediate methyl 3-amino-4-(butylamino)benzoate [24], with 1,3-benzothiazole-2-carbaldehyde in ethanol and a catalytic amount of trifluoroacetic acid at room temperature for 24 h (Supplementary Scheme S1). **OncoIr3** was obtained in a good yield (72%) using reported standard literature procedures (Supplementary Scheme S2) [25]. The chlorido-bridged dimeric iridium(III) complex [Ir(C^N)_2_(μ-Cl)]_2_ [C^N = 2-phenyl-1-[4-(trifluoromethyl)benzyl]-1*H*-benzo[*d*]imidazol-κ*N*,*C*;] [26] and the above mentioned N^N ligand in a 1:2 molar ratio served as starting materials (Supplementary Scheme S2). The obtained Ir(III) complex was characterized by ^1^H and ^13^C{^1^H} NMR spectroscopy (Fig. S4 and S5) and positive-ion HR ESI–MS (Fig. S6), showing the [M − PF_6_]^+^ signals with the expected isotopic distribution pattern. The ^1^H NMR spectrum of **OncoIr3** exhibits one set of peaks for each of the two inequivalent C^N ligands due to the asymmetric nature of the coordination environment around the metal. The new complex was shown to be at least 96% pure by both reverse‐phase RP-HPLC in DMSO (Fig. S7) and elemental analysis. The stability of **OncoIr3** in DMSO was checked by ^1^H NMR spectroscopy (Fig. S8), with no free ligands being detected after 5 days at room temperature. In addition, **OncoIr3** was stable after 24 h in biologically-relevant conditions such as in Roswell Park Memorial Institute (RPMI) culture medium as revealed by UV/Vis (Fig. S9).

The UV/Vis absorption and emission spectra of **OncoIr3** were recorded in both water (1% DMSO) and acetonitrile at room temperature (Figs. 1b and S10). The new complex displays intense absorption bands at *λ *< 340 nm that can be assigned to spin-allowed ligand-centered (^1^LC) transitions within the C^N and N^N ligands. Bands at 350–400 nm (*ε* ~ 3 × 10^4^ M^–1^ cm^–1^ at the midpoint of broad absorption band) as well as the bands in the visible range (> 400 nm) could be attributed to spin-allowed metal to ligand (^1^MLCT), spin forbidden metal-to-ligand (^3^MLCT) and ligand-to-ligand charge-transfer (LLCT) transitions [26, 27, 28]. Upon excitation at 376 nm, **OncoIr3** exhibits a broad emission band in water (1% DMSO) with emission maxima appearing at 665 nm (Fig. 1b). Importantly, the introduction of the 2-benzothiazol-yl group led to red-shift of 80 nm of the emission maximum compared to a described complex with a 2-pyridin-yl group [23] as shown in Figs. 1b and S11. This red-shift change can be explained by the electron-deficient nature of the benzothiazole moiety, leading to stabilization of the LUMO and as a consequence a smaller energy gap [29].

The emission lifetime of **OncoIr3** showed a bi-exponential decay with a very long component (1237 ns), which is consistent with excited states of triplet parentage (Table S2) [30]. Surprisingly, the emission quantum yield was higher in water (1% DMSO) than in acetonitrile in aerated solution (Table S2). This prompted us to further study emission intensity changes in a mixture of water and DMSO in different proportions. As displayed in Fig. 1c, the emission intensity increased 3 times when the water content increased from 75 to 95%. Although the excitation wavelength maxima of **OncoIr3** was 376 nm in water, the complex still maintained a large emission when it is excited at 405 nm (wavelength of excitation source used for confocal studies) (Fig. S12).

### Cell uptake and intracellular localization of OncoIr3

To check whether this new compound was able to enter human cells, cell uptake was studied by confocal microscopy and inductively coupled plasma mass spectrometry (ICP-MS).

**Fig. 2:**
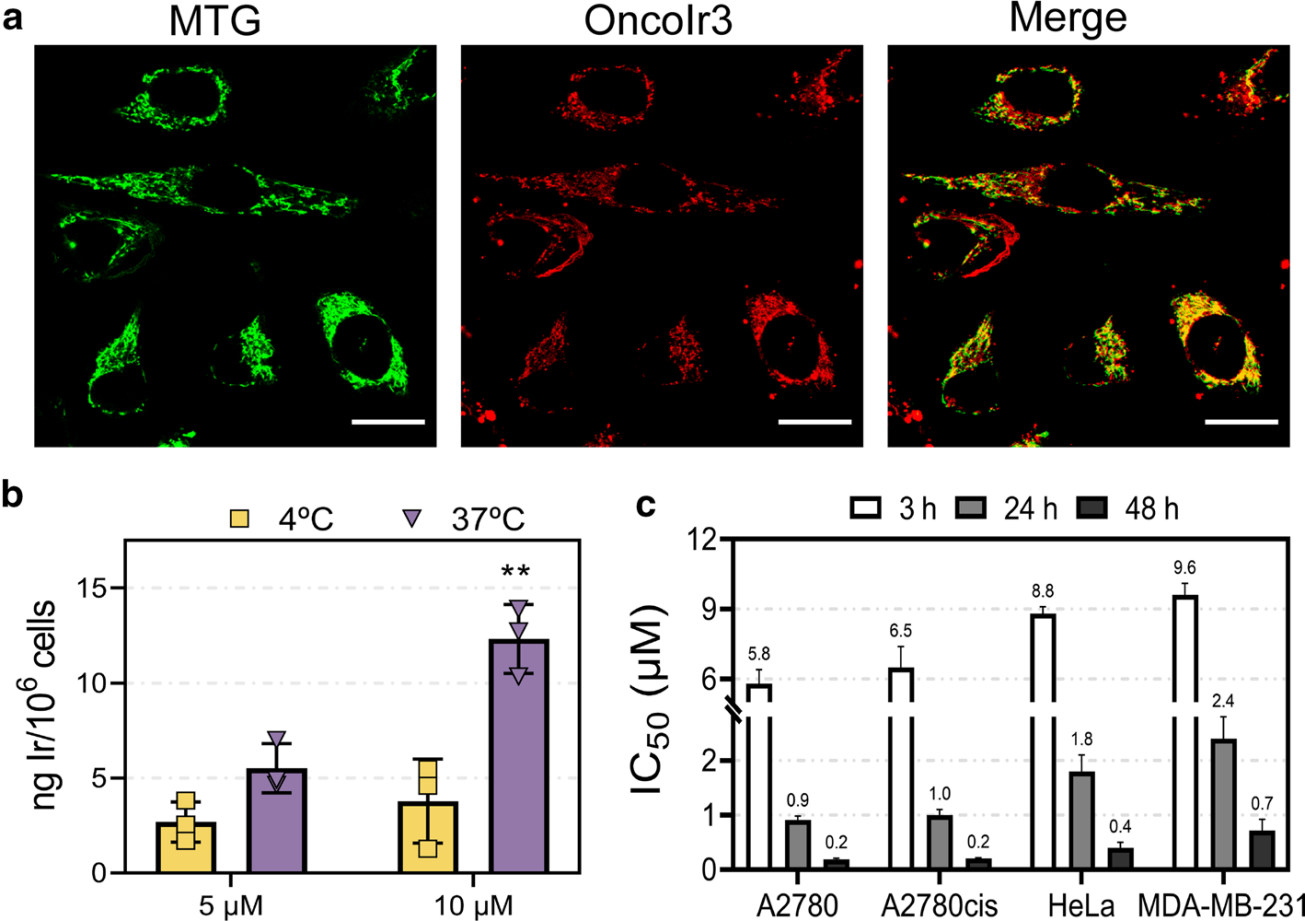
**a** Confocal images from the co-localization assay of **OncoIr3** (5 µM for 0.5 h) and Mitotracker Green (MTG; 0.1 µM for 0.5 h) in HeLa cancer cells. Scale bar = 20 µm. **b** Intracellular iridium content in A2780 cells after 2 h incubation with **OncoIr3** as determined by ICP-MS (mean ± SD from three independent experiments). Statistical significance *p < 0.05 from unpaired *t*-test. **c** Half-maximal inhibitory concentration (IC_50_) values for **OncoIr3** in cancer cell lines

As shown in Fig. S13, the fluorescence signal of **OncoIr3** was clearly observed inside A2780 and HeLa cells after 30 min at 5 µM, which confirmed rapid and excellent uptake of the compound. The pattern of staining of **OncoIr3** revealed a specific, extra-nuclear distribution in cancer cells, suggesting organelle-targeting. To investigate possible cellular targets of **OncoIr3**, a co-localization assay using the commercial mitochondrial probe MitoTracker Green FM (MTG) was performed (Figs. 2a and S14). Co-localization was measured using Pearson’s and Manders’ (M1 and M2) coefficients [31]. Our results showed a strong correlation between **OncoIr3** and MTG signals after 30 min incubation, with a Pearson’s coefficient equal to 0.672 on average. Mander’s coefficients (M1 = 0.775 and M2 = 0.936) also confirmed that **OncoIr3** was mainly located in mitochondria.

On the other hand, ICP-MS results confirmed intracellular iridium content in **OncoIr3**-treated cells (Fig. 2b). Remarkably, metal content was significantly lower in A2780 cells incubated at low temperature (4 ºC) compared to those at physiological temperature (37 ºC) after 2 h.

Strikingly, incubation for longer periods, i.e., 3 h, resulted in a change of the compound’s localization inside cancer cells (Fig. S14). A lack of overlapping with MTG was found after this period, and red phosphorescence from **OncoIr3** appeared in the form of vacuoles across the cytoplasm.

### OncoIr3 strongly inhibited cancer cell proliferation

Once demonstrated that **OncoIr3** could enter cancer cells, its antiproliferative activity was tested against a panel of human cancer cell lines including cisplatin-sensitive ovarian carcinoma (A2780) and cisplatin-resistant ovarian cancer (A2780cis), cervix adenocarcinoma (HeLa) and highly metastatic triple-negative breast cancer (MDA-MB-231). The compound was also tested in two non-tumorigenic cell lines (CHO, normal ovarian cells; BGM, normal kidney cells) to evaluate selectivity towards cancer cells. The clinical drug cisplatin, which is a well-known apoptosis inducer [32], was included for comparative purposes.

**OncoIr3** exhibited highly potent antiproliferative activities against the studied cancer cells with half-maximal inhibitory concentration (IC_50_) values within the low micromolar range after 3 h and 24 h, and nanomolar potency at 48 h (Fig. 2c and Tables S3 and S4). The highest antiproliferative activities were found in A2780 and A2780cis ovarian cancer cells. Noteworthy, **OncoIr3** was able to overcome acquired resistance mechanisms to cisplatin since similar IC_50_ values were obtained in A2780 compared to resistant A2780cis cells (resistance factors close to 1). Growth of HeLa and MDA-MB-231 cells, which display some degree of resistance to cisplatin cytotoxicity (IC_50_ > 25 µM at 48 h), was strongly inhibited by **OncoIr3**, being up to 87 times more cytotoxic than the platinum drug after 48 h (Table S3). On the other hand, the main side effect of cisplatin is nephrotoxicity. Surprisingly, the iridium complex showed no toxicity against normal renal BGM cells up to 20 µM (Table S3) and was found to be 13.7 times less toxic in normal ovarian cells CHO than in ovarian cancer cells (Table S3).

### Morphological alterations indicated oncosis induction

Since its discovery more than a century ago, oncosis has been described as a cell death mode with swelling [10]. While apoptosis is characterized by cell shrinkage and the formation of apoptotic bodies, the main feature that defines the oncotic process is cell swelling [33]. To verify that **OncoIr3** retained the oncosis-inducing ability of the parent compounds **OncoIr1** and **OncoIr2**, changes in cell morphology upon treatment were examined in first place.

**Fig. 3:**
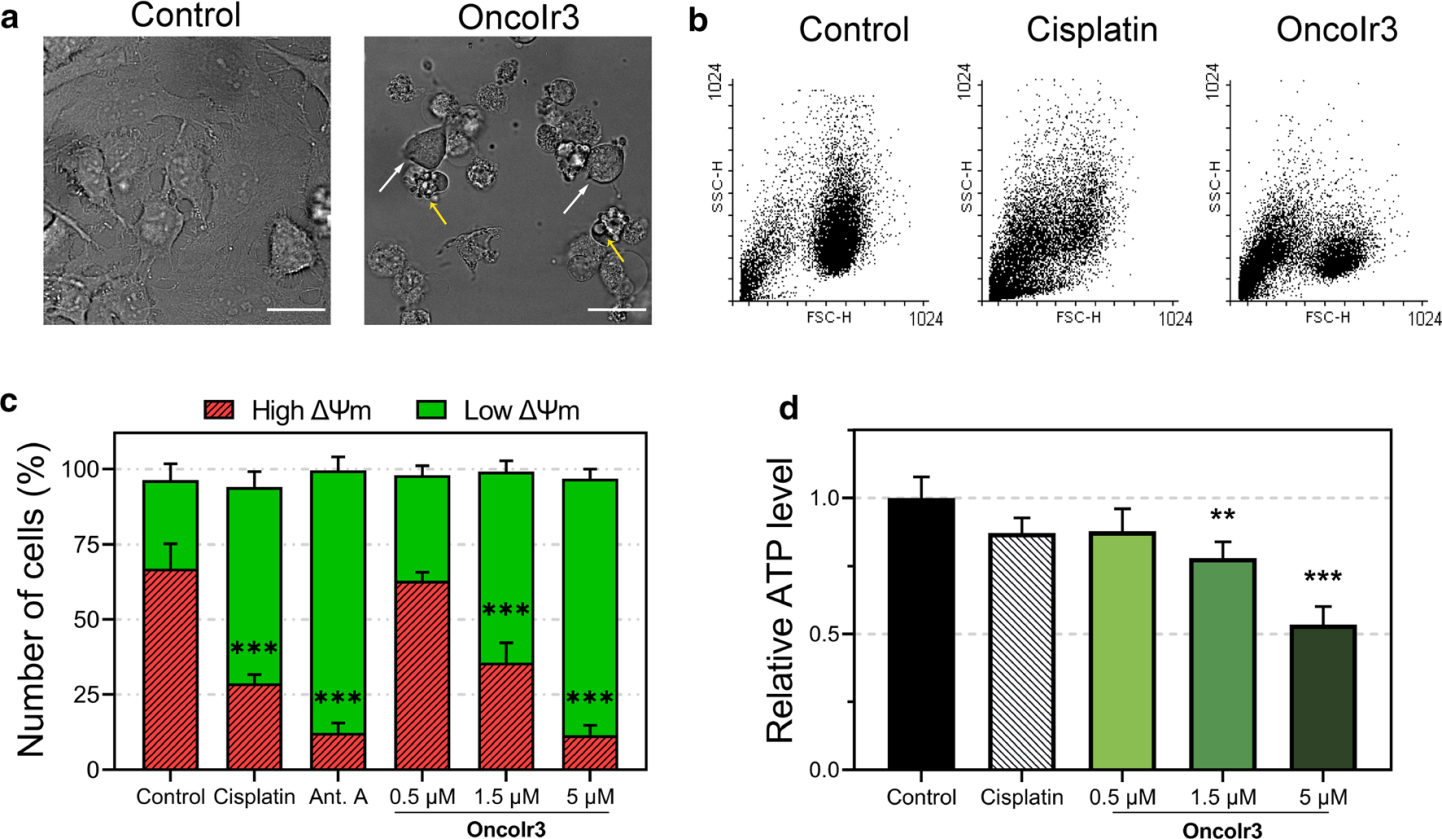
**a** Detection of cell swelling (white arrows) and cell blebbing (yellow arrows) in HeLa cells after 24 h treatment with **OncoIr3** (5 µM) Scale bar = 20 µm. **b** Cell size (FSC) vs. cell complexity (SSC) flow cytometry plots from A2780 cells after treatment with 5 µM cisplatin or **OncoIr3** for 24 h. **c** Mitochondrial membrane potential evaluation after treatment with cisplatin (5 µM), antimycin A (Ant. A; 50 µM) or **OncoIr3** determined with JC-1 dye by flow cytometry. **d** Relative levels of ATP in A2780 cells after 24 h treatment with cisplatin (5 µM) or **OncoIr3**. Statistical significance control vs. treatment *p < 0.05, **p < 0.01, ***p < 0.001 from One-Way ANOVA test

Upon treatment with **OncoIr3**, A2780 cells became rounded in shape and the number of floating cells increased after 24 h compared to cisplatin-treated A2780 cells, where apoptotic cell shrinkage was observed (Fig. S15). Nonetheless, changes in cell morphology were best detected in HeLa cells, which are bigger in size than A2780 and display defined polygonal shapes in culture. Contrary to cisplatin-induced apoptosis, which produced characteristic cell volume loss in dying cells, cellular swelling was clearly observed in HeLa cancer cells after treatment with **OncoIr3** (Fig. S15). Interestingly, the compound also compromised cell membrane since cell blebbing and detachment from culture surface were detected (Fig. 3a). The onset of these morphological alterations was consistent with oncosis induction. However, to confirm this assumption, flow cytometry assays were performed to quantify relative cell size and complexity. Forward (cell size) versus side scatter (cell complexity) plots (FSC vs SSC) analysis revealed two main populations in **OncoIr3**-treated cells: a population of swollen cells with increased FSC and another large population of small cell particles with low FSC/SSC ratios corresponding to dead cells (Fig. 3b). In contrast, cells treated with an apoptosis-inducing agent such as cisplatin showed a plot profile with a transient increase in SSC and a concomitant decrease in FSC populations, which are indicative of cell shrinkage by apoptosis (Fig. 3b). Increases in average cell size were also detected on FSC over time after treatment with **OncoIr3** (Fig. S16).

### OncoIr3 caused mitochondrial dysfunction, generation of reactive oxygen species (ROS) and ATP depletion

Mitochondria play a central role during cell death initiation [34]. In oncosis, energetic failure leads to mitochondrial dysfunction and produces a dramatic reduction of ATP production, which results in plasma membrane leakage [35]. Therefore, a series of experiments were performed to monitor mitochondrial function of cancer cells upon **OncoIr3** treatment.

First, mitochondrial membrane potential (ΔΨm) dissipation was investigated in A2780 cells using JC-1 dye, a probe that shows changes in the red to green fluorescence signal ratio depending on the degree of ΔΨm depolarization. A decrease in red to green fluorescence ratio was detected upon increasing concentrations of **OncoIr3,** indicating a dose-dependent loss of ΔΨm (Figs. 3c and S17).

In addition, since ATP synthesis requires functional mitochondria, we measured intracellular ATP content upon treatment with **OncoIr3**. The intracellular ATP levels of **OncoIr3**-treated cells experienced a pronounced dose-dependent decrease, with a 47% reduction with 5 μM compared to untreated cells (Fig. 3d).

To test whether this ΔΨm depletion was related to ROS generation [36], the fluorescent probe dihydroethidium (DHE) was used. After 6 h incubation with **OncoIr3**, an increase in red fluorescence from DHE probe was observed in A2780 cells upon 5 μM treatment (Fig. S18). A dose-dependent increase trend in ROS levels was also found upon increasing concentrations of **OncoIr3** after 3 h and 6 h (Fig. S18).

Altogether, these experiments demonstrated that **OncoIr3** caused mitochondrial dysfunction that was characterized by loss of ΔΨm, ATP depletion and ROS production.

### Treatment with OncoIr3 provoked cell membrane injury

**Fig. 4:**
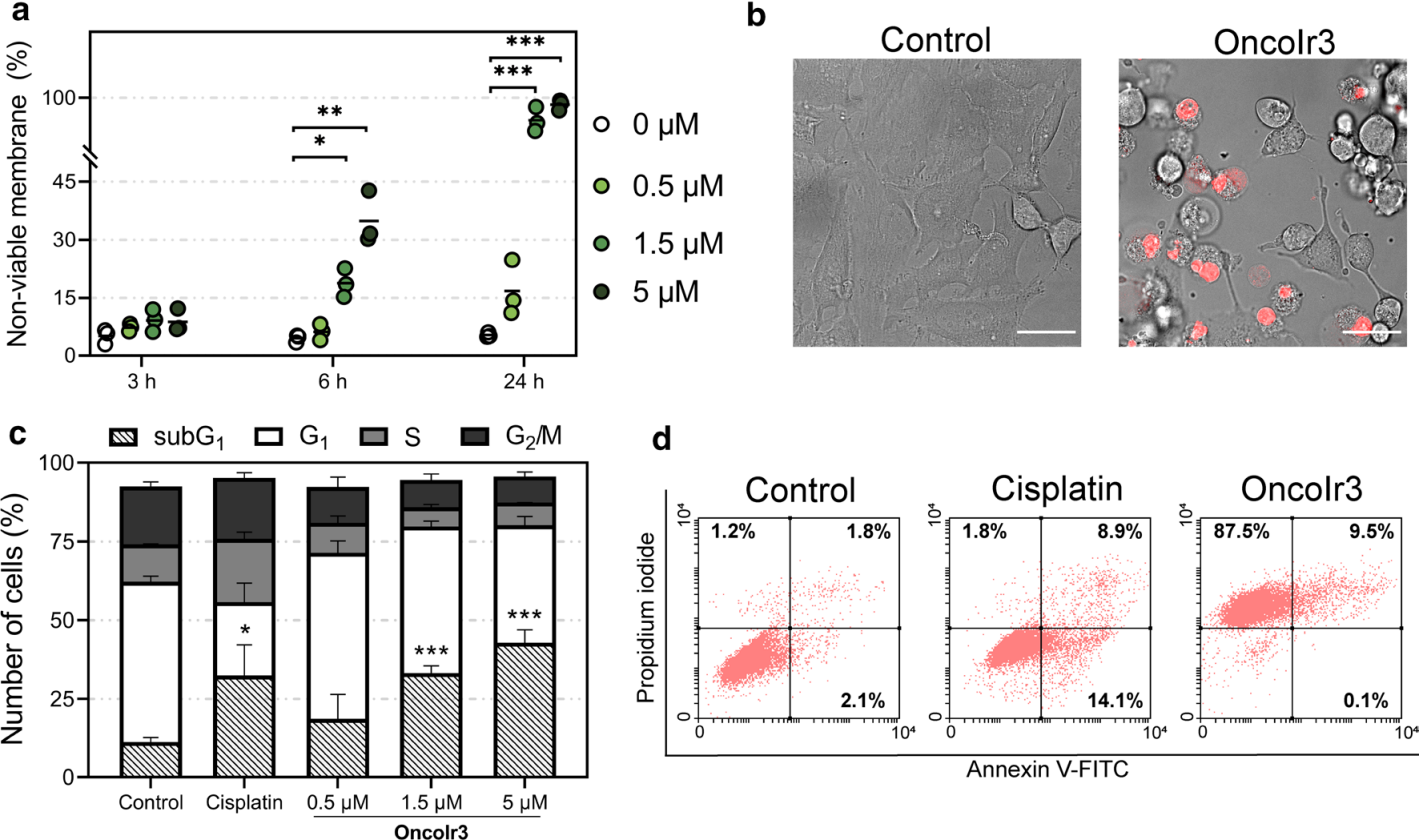
**a** Membrane integrity test of A2780 cells determined by propidium iodide entry after treatment with **OncoIr3** (*p < 0.05, ** *p* < 0.01, ***p < 0.001; unpaired *t* test). **b** Detection of cell membrane rupture and permeabilization in cancer cells following **OncoIr3** (5 µM) treatment by fluorescence microscopy using propidium iodide staining (red). Scale bar = 20 µm. **c** Percentage of A2780 cells (mean ± SD from three independent experiments) in sub-G_1_, G_1_, S, and G_2_/M phases of the cell cycle after treatment with cisplatin (5 µM) or **OncoIr3** (*p < 0.05, ***p < 0.001; unpaired *t* test). **d** Representative flow cytometry dot plots of A2780 cells stained with Annexin V-FITC/Propidium iodide labelling method after treatment with cisplatin or **OncoIr3** (5 µM) for 24 h

As oncosis cell death progress, energy-dependent pumps in plasma membrane become unable to maintain ionic homeostasis and cell membrane permeabilization eventually occurs [10, 37]. Permeability of plasma membrane can be detected by dye exclusion test. We used propidium iodide, a fluorescent intercalating agent that can permeate into cells with compromised membranes, to evaluate the impact of **OncoIr3** on cell membrane integrity. As shown in Figs. 4a and S19, A2780 cells treated with **OncoIr3** allowed significant propidium iodide entrance after 6 h at 1.5 μM and 5 μM. Incubation for a longer period (24 h) resulted in cell populations without viable plasma membrane (Figs. 4a and S19). This rupture of cell membrane was further confirmed by fluorescence microscopy after propidium iodide staining (Fig. 4b).

### Cell death induced by OncoIr3 led to DNA breakdown and necrosis

To gain insights into the mechanism underpinning **OncoIr3**, additional cell death studies were performed. On one hand, the impact of the compound on cell cycle distribution was examined in A2780 cancer cells. Flow cytometry analysis showed that **OncoIr3** produced a cell cycle arrest in G_1_ phase and significantly increased subG_1_ cell populations, an indication of DNA breakdown (Figs. 4c and S20) [38]. In contrast, the well-known DNA damaging agent cisplatin induced S phase arrest along with a subG_1_ peak (Figs. 4c and S20). On the other hand, flow cytometry analyses using dual Annexin V-FITC/Propidium iodide (AV/PI) staining were carried out. These assays allow the identification of early and late apoptosis in AV^+^/PI^−^ and AV^+^/PI^+^ regions, respectively, as well as necrosis as AV^−^/PI^+^. Unlike cisplatin, which mainly induced AV^+^ cells, treatment with **OncoIr3** induced large AV^−^/PI^+^ populations after 24 h and negligible AV^+^ cell populations (Figs. 4d and S21). Accumulation of AV^−^/PI^+^ cells upon treatment with **OncoIr3** was found to be time-and dose-dependent (Fig. S22–S24).

In addition, cell death survival upon pretreatment with caspase-dependent apoptosis (NSCI, 5 μM) [39], p53-dependent apoptosis (pifithrin-alpha, 5 μM) [40], and paraptosis (cycloheximide, 50 μM) [41] inhibitors was measured. However, these inhibitors did not rescue A2780 cancer cells from **OncoIr3**-induced cell death as cell viability was found to be comparable with and without pre-treatments (Fig. S25a). To discriminate between major forms of necrosis, two inhibitors, necrostatin-1 and leupeptin, which cause necroptosis and lysosomal protease inhibition, respectively, were also employed in survival assays. Nonetheless, the response in the pretreatments with these inhibitors showed no increase in cell viability (Fig. S25b).

Since cell cycle arrest can contribute to anti-migration and anti-metastatic properties of chemical compounds, wound-healing assays were also performed. Well-defined wounds were created in confluent cell layers and the ability of cancer cells to migrate through the gap was examined under microscopy. As shown in Fig. S26, incubation with **OncoIr3** resulted in a strong inhibition of cell migration between the two initially separated cell compartments as compared with the untreated control group.

### Development of a *Caenorhabditis elegans* tumoral model for testing OncoIr3 in vivo

Owing to the potency of **OncoIr3** observed in vitro, we decided to test the antitumor activity of the compound in vivo. The nematode *Caenorhabditis elegans* was used to develop a tumoral model. These animals offer great advantages for preclinical chemotherapeutic screening due to short generation times, and homogenous populations with a large number of individuals [42].

**Fig. 5:**
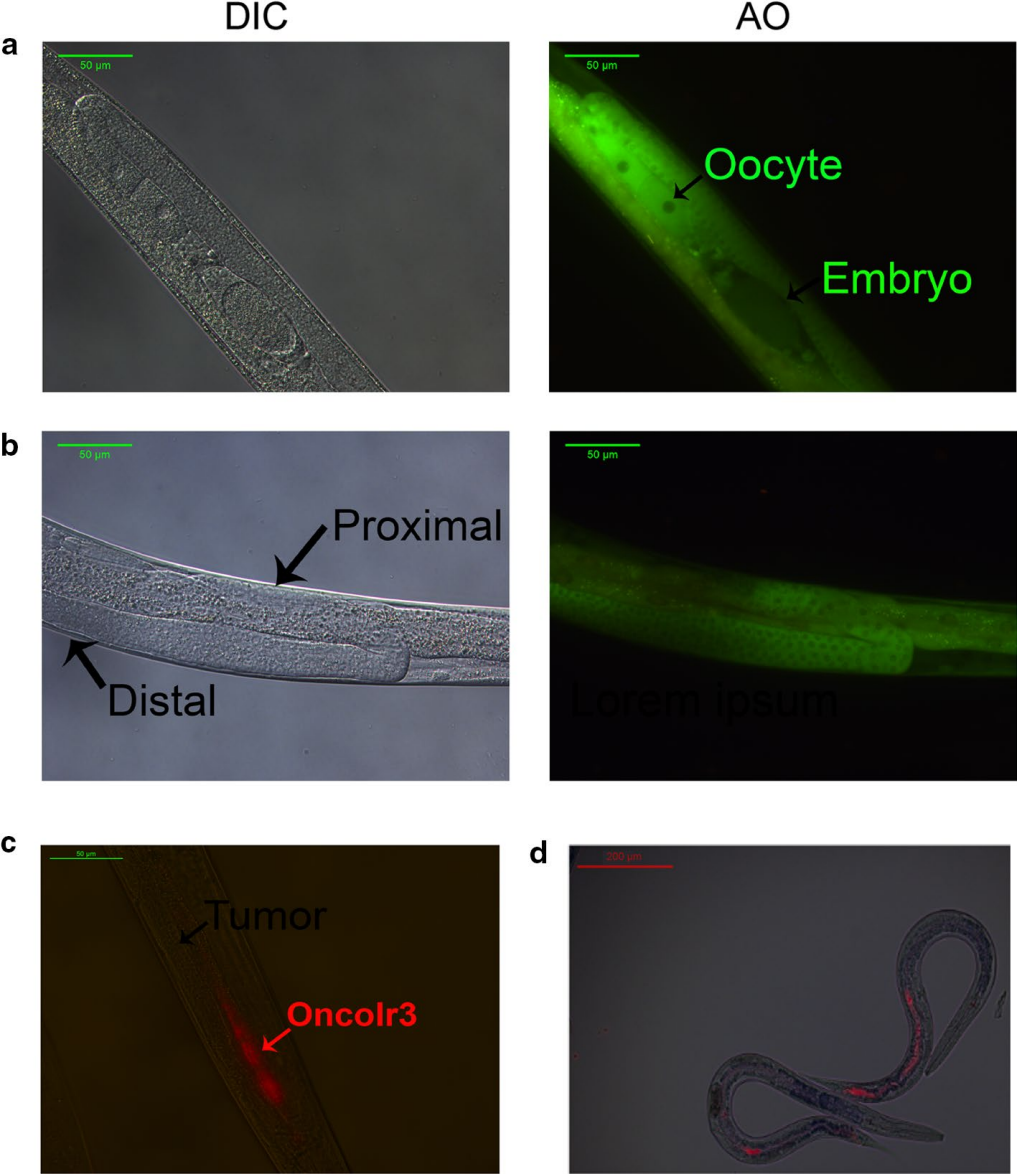
**a** Wild-type gonads of the *C. elegans* strain N2 visualized with differential interference contrast (DIC) and stained with acridine orange (AO). Scale bars 50 μm. **b** Tumoral gonads of the *C. elegans* strain JK1466. Scale bars 50 μm. **c**, **d** Detection of **OncoIr3** inside tumoral JK1466 nematodes Scale bar: 200 μm

Since ovarian cancer cells were strongly inhibited by **OncoIr3**, a germ cell tumor was induced in *C. elegans*. Using RNAi technology, the *gld-1* gene was silenced [43]. *gld-1* is a tumor suppressor gene involved in *C. elegans* oocyte development and it is necessary for mitosis and meiosis regulation in germline cells [43]. In *gld-1(q485)* mutants (JK1466 strain), germ cells fail to exit from mitosis. This causes a sustained proliferation that eventually forms large tumors in the proximal gonad which are lethal to the animals [44]. In Fig. 5a, gonads of normal young adults are shown, with oocytes and embryos in the proximal zone and germinal cells in the distal zone. In contrast, tumoral strain nematodes showed proliferative germ cells in all the gonad regions and the absence of oocytes and embryos (Figs. 5b and S27).

Prior to in vivo antitumor evaluation, assimilation of **OncoIr3** by the nematodes was first investigated. Animals were supplied with the compound along with *Escherichia coli* as a standard food source. Owing to its bright phosphorescence, **OncoIr3** was detected inside animals in the intestinal lumen (Fig. 5c). The compound was also visible in the pharynx and in the grinder (Fig. 5d). Furthermore, a strong correlation between compound assimilation and dosage supplied was noted, indicating a dose-dependent behavior (Fig. S28).

### OncoIr3 effectively reduced tumoral growth in *C. elegans* and extended their lifespan

**Fig. 6:**
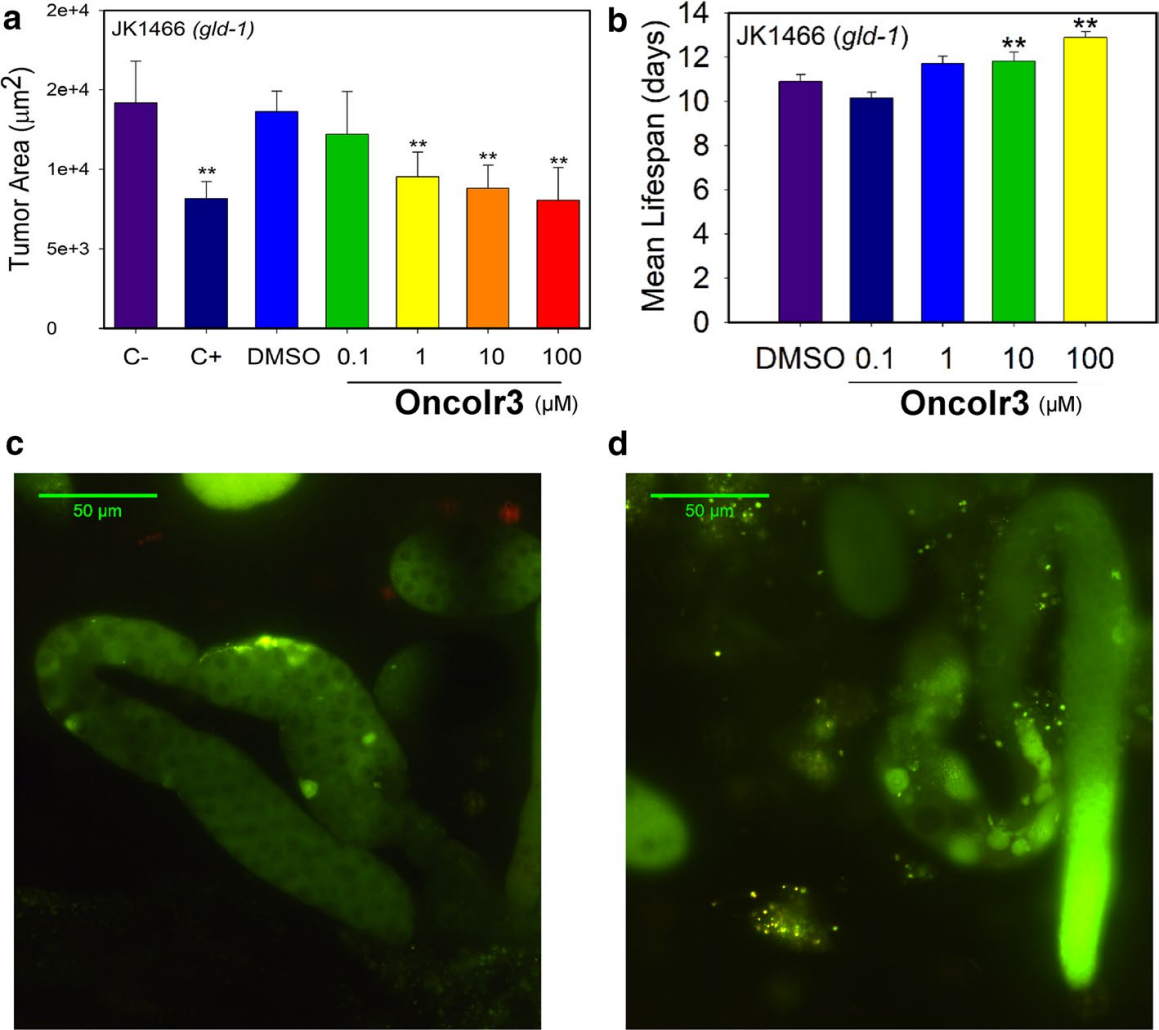
**a** In vivo tumor size evaluation upon **OncoIr3** treatment. Data are represented as mean ± S.D (*n* = 10 per treatment group), **significantly different at *p* ≤ 0.05 by ANOVA test. **b** Mean lifespan of JK1466 strain treated with different concentrations of **OncoIr3**. Data are represented as mean lifespan ± S.E, **significantly different at *p* ≤ 0.05 by Log Rank test. **c**, **d** Representative images of extracted gonads of tumoral animals stained with acridine orange and treated with 100 μM of cisplatin (**c**) or with **OncoIr3** (**d**). Scale bars: 50 μm

At this point, the antitumor efficacy of **OncoIr3** was evaluated. Synchronized JK1466 tumoral worms were treated with the compound at different concentrations (0.1, 1, 10 and 100 µM), and the tumor area was measured after four days. Strikingly, low doses of **OncoIr3**, i.e., 1 µM, were sufficient to significantly decrease tumor area in the nematodes, denoting a highly potent antitumor activity in vivo (Fig. 6a). Measurements of tumor growth revealed that **OncoIr3** strongly reduced the size of tumors in a dose-dependent manner (Fig. 6a and Table S5). Remarkably, **OncoIr3** at 10 and 100 μM led to a significant decline in tumor area of 35.45% and 41.03% (*p* < 0.0001), respectively, compared to untreated animals (Table S5).

Additionally, we evaluated the lifespan of model animal *C. elegans* by means of survival assays for both wild-type and tumoral strains. The measurement of lifespan was carried out using the automatic lifespan machine, which provides visual record of individual deaths automatically and allows construction of survival curves over the whole lifespan of animals [19]. Our results showed that **OncoIr3** significantly increased the lifespan of the tumoral animals, with an increment of 18.4% (*p* ≤ 0.05) at 100 µM (Fig. 6b, Table S6 and Fig. S29). Interestingly, treatment with the compound did not affect lifespan of wild-type individuals, indicating minimal toxicity toward the animals (Fig. S29). Noteworthy, animal deaths were delayed until 11^th^ day under 100 µM treatment with **OncoIr3** (Fig. S29).

Further experiments were performed to explore the toxicity of **OncoIr3.** Wild-type animals were exposed to higher concentrations of the compound, namely 0.1, 0.5 and 2 mM for 48 h to evaluate possible harmful effects. *C. elegans* development, lethality and motility were monitored because these are endpoint parameters that have shown good correlation with acute toxicity in rodents [45]. Measurements of animal’s body length showed no significant difference between the control nematodes and the **OncoIr3**-treated ones (Fig. S31E). Moreover, the development was normal in all the conditions as shown in the representative images of Fig. S31 A-D. As expected for specimens grown at 20 ºC for 48 h, the animals had developed from L1 larvae to L4 larvae stage. In this stage, the vulva is not yet totally developed, and the nematodes lack oocytes and eggs. The same treated cohorts were transferred to clean NGM plates to study **OncoIr3** lethality. No dead animals were scored after 48 h exposition to high concentrations of the novel compound. As demonstrated in the Supplementary Video 1, the animals moved freely when they were stimulated with the macroscope light. Furthermore, worms’ locomotion was also within the normal values (Fig. S32), and there was no significant change in maximum speed as shown in the normal distribution curves (Fig. S32A). Interestingly, the average speed of the *C. elegans* exposed to 100 µM of **OncoIr3** was slightly higher than in the control animals, 0.25 and 0.17 mm s^−1^, respectively (Fig. S32B, C). In any case, both values fall within the normal range for the worms 0.109–0.35 mm s^−1^ [46, 47, 48]. Motility patterns obtained from Supplementary Video 1 (Fig. S32D), suggest a normal disperse behavior that occurs when *C. elegans* are removed from bacterial lawn.

### Oncosis was detected in vivo after OncoIr3 treatment

Next, we used acridine orange staining in our developed *C. elegans* tumoral model to interrogate whether oncosis mode of cell death was also induced in vivo. This staining allowed the visualization of the individual cells in the gonad and their morphology. Acridine orange can only penetrate into cell nuclei if nuclear membrane is compromised and emits fluorescence when bound to DNA. As observed in Fig. 6c, the gonad cells of the animals treated with cisplatin exhibited small fluorescent corpses corresponding to typical apoptotic bodies. In contrast, gonads from **OncoIr3**-treated animals showed a completely different pattern, with swollen and enlarged cells which are properties associated to oncosis (Fig. 6d).

In addition, the ability of **OncoIr3** to generate ROS was also tested in vivo. DHE staining revealed a slight increase in ROS levels in the nematodes upon treatment with the compound, which corroborated ROS involvement in the **OncoIr3**-mediated cell death (Fig. S30).

## Discussion

Tumor cells often exhibit defective apoptotic pathways [49]. These defects not only imply sustained tumor proliferation, but also render resistance to chemotherapeutic treatments [1]. Given the burden of this problem, we were inspired to prepare a novel compound with the ability to induce apoptosis-independent cell death.

By redesigning the molecular core of **OncoIr1** and **OncoIr2**, we synthesized **OncoIr3**, a fine-tuned iridium metallodrug with advantageous photophysical properties and improved cellular uptake compared to parent compounds (Fig. 1). **OncoIr3** rapidly penetrated the cancer cells via energy-dependent mechanisms and targeted mitochondria (Fig. 2), although after 3 h, its location changed to perinuclear areas, which suggested vacuolization from lysosomes [50]. Once internalized, **OncoIr3** not only showed improved anticancer activity over cisplatin in all tested cell lines, but also displayed equipotent cytotoxicity toward Pt-resistant A2780cis cancer cells (Fig. 2). The ability to overcome drug resistance mechanisms suggested a mode of action different than that of platinum drugs, which usually induce apoptosis [51]. Indeed, cell morphology analysis revealed non-apoptotic cellular features, such as cell swelling and blebbing, compromised plasma membrane and increased cell volume (Fig. 3). The onset of these morphological alterations matched with oncosis induction.

Oncosis has been postulated to progress in three phases: an initial stage where membrane injury and ATP depletion leads to cell swelling, a following stage involving loss of cell membrane integrity, and a third phase showing necrotic features [9]. Apoptosis is in many ways the opposite: an ATP-dependent process characterized by cell shrinkage [52]. We found that **OncoIr3** reduced ATP levels and decreased ΔΨm in a concentration-dependent manner (Fig. 3). ROS generation might also be involved in **OncoIr3** mechanism, probably due to mitochondrial targeting (Fig. S18). The ΔΨm collapse would then effectively lead to depletion of cell energy stores, which in turn results in leak of water and ions through plasma membrane. In effect, this is what we observed during propidium iodide exclusion tests. Cells became permeant to the dye in a dose-dependent manner after 6 h due to cell membrane injuries that are ascribable to second stage of oncosis (Figs. 3 and S19). After 24 h, total disruption of cell membrane was detected, indicating entry into necrotic phase (Figs. 3, S19 and S20).

Certain biochemical features of apoptosis such as phosphatidylserine translocation were largely absent during **OncoIr3**-elicited cell death (Figs. 4 and S21–S24). However, DNA breakdown, which is another characteristic of apoptotic induction [53], was appreciated after 24 h (Fig. 4). This led us to investigate a possible crosstalk between apoptosis and oncosis. Neither caspase inhibitor nor p53 inhibitor reduced **OncoIr3** cytotoxicity, suggesting that caspase- and p53-dependent apoptotic activation did not play important roles in its mechanism of action (Fig. S25a). Paraptosis, a cell death mode that requires protein synthesis, did not seem to be contributing either. These findings pointed out a necrosis-related mechanism. To further investigate this, necrotsatin-1 and leupeptin were used to inhibit either necroptosis or lysosomal proteases such as calpains, which have been associated to oncotic cell death [54]. However, we discovered that none of these pharmacological inhibitors prevented **OncoIr3**-elicited cell death, suggesting that the compound might not require neither necrosome formation nor lysosomal proteases to produce its oncotic anticancer activity (Fig. S25b). The unique mode of action of **OncoIr3** also involved potent anti-migration properties in vitro, which could be of interest to block cancer metastasis (Fig. S26).

Our next step was to validate the anticancer activity of **OncoIr3** in vivo. The nematode *C. elegans* was chosen for this purpose. Tumor induction in *C. elegans* was achieved via gene knockdown with RNAi technology, thereby producing a tumoral phenotype for anticancer screening [55]. It is worth noting that 83% of *C. elegans* genes have human homologous genes [56]. *C. elegans* have also gustatory and olfactory receptors to detect food, danger or other individuals; the responses to chemosensory stimuli producing behavioral changes highly conserved across animal kingdom [57, 58]. We found that administration of **OncoIr3** was not repelled by the nematodes as it happens with other chemical compounds such as heavy metals or alkaloids [59]. In fact, **OncoIr3** was assimilated via active ingestion by the worms (Fig. 5). Importantly, the fact that mean lifespan of the tumoral nematodes was extended by **OncoIr3** treatment while lifespan of wild-type animals remained unaffected suggested that longevity promotion was due to tumoral growth reduction. Indeed, increasing the concentration of **OncoIr3** up to 20-fold the effective dose (2 mM) did not result in any negative effects on key endpoint parameters such as development, lethality and motility, thus indicating low toxicity and minimal adverse effects associated to this compound (Fig. S31, 32). Finally, the detection of oncosis induction in the animals corroborated the non-apoptotic mode of action of **OncoIr3** (Fig. 6).

Altogether, we believe that this combination of in vitro and in vivo screening using cell- and *C. elegans*-based models provides a promising workflow to discover and validate anticancer drugs that act via non-conventional mechanisms. Although these models could not replace mammalian testing, they significantly reduce animal tests, thus leading to economical savings, and provide useful information not only on potential application to humans, but also on the underlying mechanism of action of the tested compound [60]. Owing to the high anticancer drug potential and good safety profile in *C. elegans,* the preclinical data herein obtained makes **OncoIr3** an ideal candidate for further drug development with promise for apoptosis-refractory cancers.

In summary, a novel iridium(III) metallodrug **OncoIr3** with interesting bioimaging and anticancer properties has been synthesized and characterized. The compound exhibits potent anticancer activity in vitro against a panel of cancer cells and in vivo in a developed *C. elegans* tumoral strain. The main mechanism of action of **OncoIr3**, which has been deeply characterized in this study, is related to oncosis, a relatively less explored cell death mode compared to apoptosis. This metal-based agent could serve as lead compound for the development of a new class of oncosis-inducing anticancer compounds.

## Supplementary Information

Below is the link to the electronic supplementary material.

Supplementary file1 (DOCX 8897 KB)

Supplementary file2 (MP4 4816 KB)

## Data Availability

The datasets generated during and/or analysed during the current study are available from the corresponding author on reasonable request.
