# Supplementary material for: Potent anticancer activity of a novel iridium metallodrug via oncosis
Source: Cell Mol Life Sci. 2022 Sep 6;79(10):510. doi: 10.1007/s00018-022-04526-5 (PMC9448686; doi:10.1007/s00018-022-04526-5)
Supplement: Supplementary file 1 — Supplementary file1 (DOCX 8897 KB) [file 18_2022_4526_MOESM1_ESM.docx]

Supplementary information

**Potent anticancer activity of a novel iridium metallodrug *via* oncosis**

Enrique Ortega-Forte ^a+^, Samanta Hernández-García ^b+^, Gloria Vigueras ^a+^, Paula Henarejos-Escudero ^b^, Natalia Cutillas ^a^, José Ruiz ^a*^, Fernando Gandía-Herrero ^b*^

^a^ Departamento de Química Inorgánica. Universidad de Murcia, and Institute for Bio-Health Research of Murcia (IMIB-Arrixaca), E-30071 Murcia, Spain.

^b^ Departamento de Bioquímica y Biología Molecular A. Unidad Docente de Biología, Facultad de Veterinaria, Universidad de Murcia.E-30071 Murcia, Spain.

+ These authors contributed equally to this work.

* Corresponding authors: Fernando Gandía-Herrero [fgandia@um.es](mailto:fgandia@um.es) and José Ruiz [jruiz@um.es](mailto:jruiz@um.es)

Table of contents

[1. Synthesis and Characterization 3](#_Toc100665686)

[2. Stability and purity studies 8](#_Toc100665687)

[3. Photophysical properties 11](#_Toc100665691)

[4. *In vitro* biological evaluation 14](#_Toc100665692)

[5. *In vivo* biological evaluation 26](#_Toc100665695)

**Supplementary Methods**

# Synthesis and Characterization

- 1. ***General reagents used in the synthesis and instrumentation***

Starting materials: IrCl_3_•H_2_O (Johnson Matthey) was used as received. 2-phenylbenzimidazole, 1,3-benzothiazole-2-carbaldehyde, trifluoroacetic acid and magnesium sulfate were obtained from Sigma-Aldrich (Madrid, Spain) and used without further purification. Methyl 3-amino-4(butylamino)benzoate was synthesized as previously described [1]. The HC^N proligand was synthesized as previously reported [2]. Deuterated solvents were purchased from Euriso-top. All synthesis procedures were carried out under nitrogen atmosphere conditions using standard Schlenk techniques. The purity of all biologically evaluated molecules, based on HPLC and elemental analysis, is > 95 %.

The C, H, and N analyses were performed with a Carlo Erba model EA 1108 microanalyzer. The ^1^H and ^13^C spectra were recorded on a Bruker AC 300E, Bruker AV 400, or Bruker AV 600 NMR spectrometer and chemical shifts are cited relative to SiMe_4_ (^1^H and ^13^C, external). ESI mass (positive mode) analyses were carried out on an HPLC/MS TOF 6220. The isotopic distribution of the heaviest set of peaks matched very closely with that calculated for the formulation of the complex cation.

- 1. ***Synthesis of N^N ligand methyl 2-(benzo[d]thiazol-2-yl)-1-butyl-1H-benzo[d]imidazole-5-carboxylate.***

The method was adapted from the previously described [1]. Methyl 3-amino-4- (butylamino)benzoate (1.12 mmol, 250 mg) and 1,3- benzothiazole-2-carbaldehyde (1.12 mmol, 183 mg) were dissolved in ethanol (7 mL) in a round-bottom flask. Then, trifluoroacetic acid (0.11 mmol, 53 μL) and magnesium sulfate (5.56 mmol, 670 mg) were added and the reaction was stirred at room temperature for 24 h. The mixture was filtered to remove magnesium sulphate and the filtrate was concentrated and dissolved in dichloromethane. Dichloromethane was extracted with water (2 × 10 mL) and brine (10 mL), dried on magnesium sulfate, and concentrated under reduced pressure. The brown solid was washed with hexane. Isolated yield: 32 %.^1^H-NMR (400 MHz, CDCl_3_): δ 8.77 (d, J=0.8 Hz, 1H), 8.21 (dd, J=8.8 Hz, 1.6 Hz, 1H), 8.17 (m, 1H), 8.05 (m, 1H), 7.60 (m, 3H), 5.06 (t, J=7.6 Hz, 2H), 3.98 (s, 3H), 2.00 (m, 2H), 1.50 (m, 2H), 1.03 (t, J= 7.2 Hz, 3H). ^13^C{^1^H} NMR (100 MHz, CDCl_3_): δ 168.37, 160.11, 155.07, 147.63, 143.41, 140.74, 136.50, 127.53, 127.41, 126.62, 126.35, 125.04, 124.07, 122.80, 111.10, 53.21, 46.38, 33.08, 21.08, 14.76. ESI-MS (pos. ion mode, CH_3_CN): m/z = 366.1278 [M+H]^+^. Anal. Calcd. for C_20_H_19_N_3_O_2_S: C, 65.73; H, 5.24; N, 11.50; S, 8.77. Found: C, 65.5; H, 5.0; N, 11.3; S, 8.8 (%).

- 1. ***Synthesis of OncoIr3***

The complex was synthesized with a slight modification of the standard literature procedures for other Ir(III) complexes [3,4].

First, the cyclometalated iridium(III) chloro-bridged dimer was obtained by reaction of 2-phenyl-1-[4-(trifluoromethyl) benzyl]-1H-benzo[d]imidazole (2.2 mmol) and iridium(III) chloride (1 mmol) in 2-ethoxyethanol/deionized H_2_O (3:1) in a Schlenk flask at 110 ºC for 24 h under a nitrogen atmosphere. The resultant solid was collected by filtration and washed with water and ethanol. Then, the dimer (1.0 mmol) and the N^N ligand (2.1 mmol) were dissolved in dichloromethane/methanol (2:3, v/v) in a Schlenk flask. The mixture was stirred at 58 ºC for 24 h under a nitrogen atmosphere. After cooling the solution to room temperature, an excess amount of KPF_6_ (2.5 mmol) was added, and the mixture was stirred for 30 min. The solvent was removed under reduced pressure, and the solid was washed with water. The product was recrystallized from dichloromethane and ether.

Red solid. Isolated yield: 72 %. ^1^H-NMR (300 MHz, DMSO-d_6_): δ 8.47 (d, J = 7.9 Hz, 1H), 8.18 (d, J = 8.8 Hz, 1H), 8.05 (dd, J = 8.8, 1.5 Hz, 1H), 7.85 (d, J = 8.0 Hz, 1H), 7.79-7.73 (m, 3H), 7.67-7.61 (m, 1H), 7.50 (d, J = 8.2 Hz, 2H), 7.32 (d, J = 8,3 Hz, 2H), 7.23-7-18 (m, 3H), 7.13-7.06 (m, 3H), 7.05-6.76 (m, 8H), 6.73 (d, J = 1.0 Hz, 1H), 6.49 (dd, J = 7.6, 1.2 Hz, 1H), 6.37 (dd, J = 7.7, 0.9 Hz, 1H), 6.31-6.15 (m, 4H), 5.80 (d, J = 8.2 Hz, 1H), 5.69 (d, J = 8.2 Hz, 1H), 5.02-4.90 (m, 2H), 3.52 (s, 3H), 1.90-1.72 (m, 2H), 1.21-1.09 (m, 2H), 0.69 (t, J = 7.2 Hz, 3H). ^13^C- NMR (150 MHz, DMSO-d_6_): δ 166.24, 164.27, 163.74, 158.83, 152.68, 151.96, 151.04, 147.32, 142.21, 140.57, 139.77, 139.56, 139.32, 136.49, 136.42, 135.61, 135.26, 135.11, 134.78, 134.34, 131.70, 131.58, 130.03, 129.83, 129.35, 128.25, 128.05, 127.95, 127.63, 127.29, 127.07, 126.73, 126.02, 125.75, 125.23, 124.07, 123.75, 123.03, 121.35, 114.89, 114.76, 114.61, 113.33, 113.20, 52.92, 48.08, 47.77, 33.37, 20.72, 14.81. ESI-MS (pos. ion mode, DMSO): m/z = 1260.3054 [M - PF_6_]^+^. Anal. Calcd. for C_62_H_47_F_12_IrN_7_O_2_PS: C, 52.99; H, 3.37; N, 6. 89; S, 2.28 Found: C, 53.01; H, 3.35; N, 6.87; S, 2.29 (%).


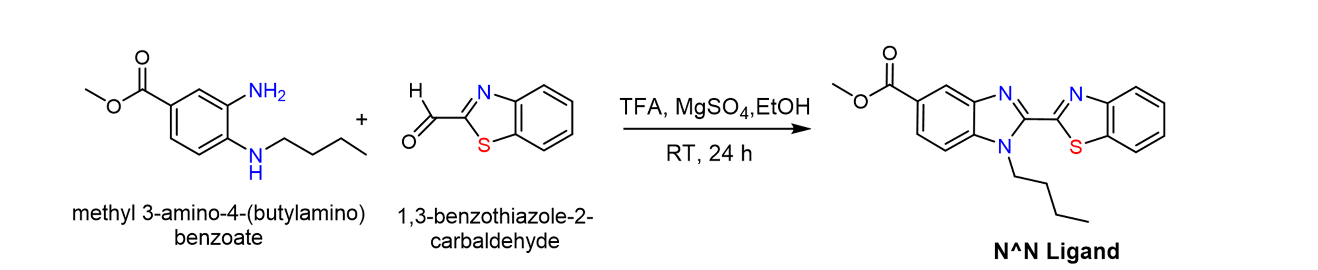


**Scheme S1.** Synthesis of N^N ligand.

**Scheme S2**. Synthesis of **OncoIr3** complex.

 **Fig. S1.** ^1^H NMR in CDCl_3_ for N^N ligand.


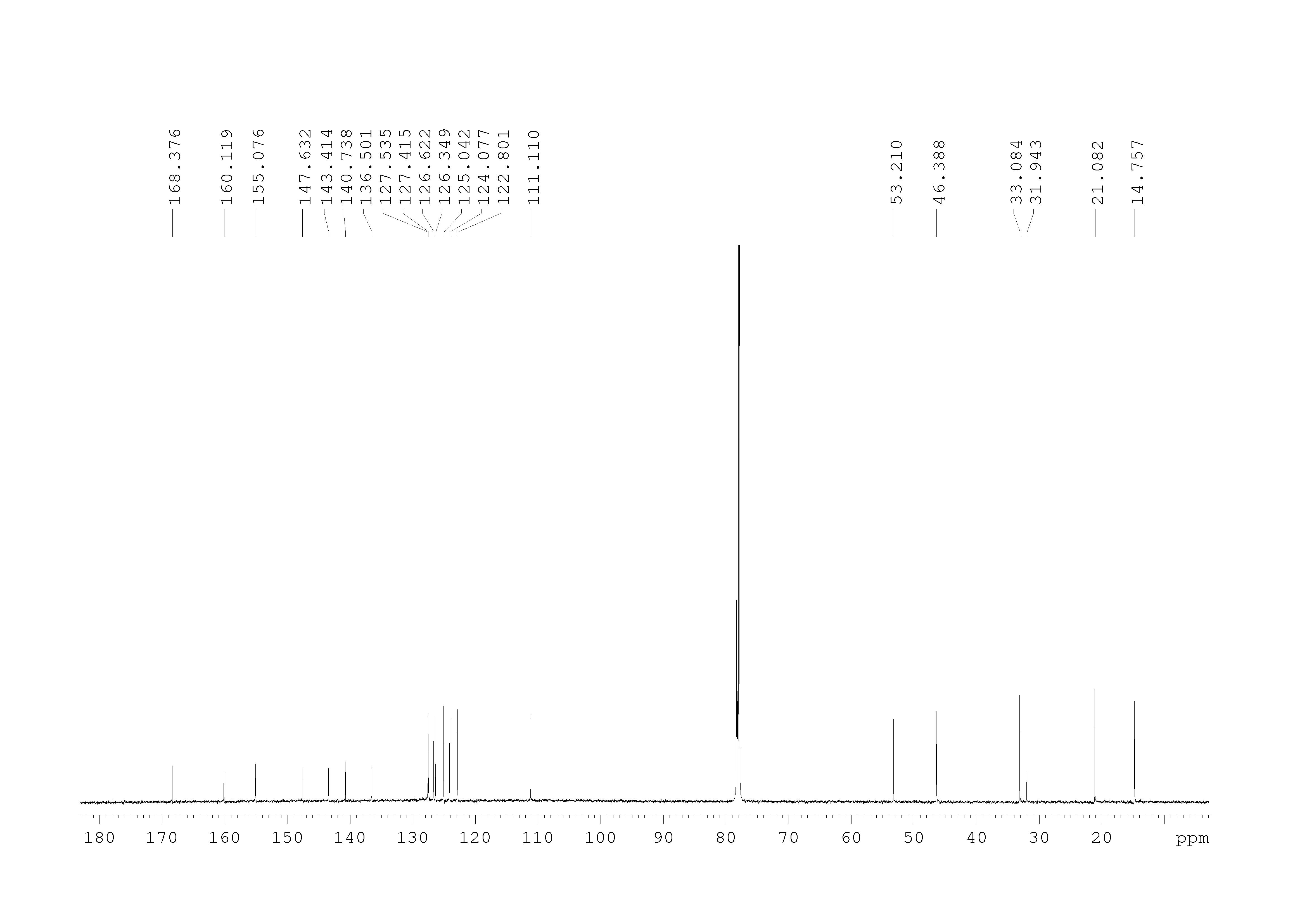


**Fig. S2.** ^13^C NMR in CDCl_3_ for N^N ligand.


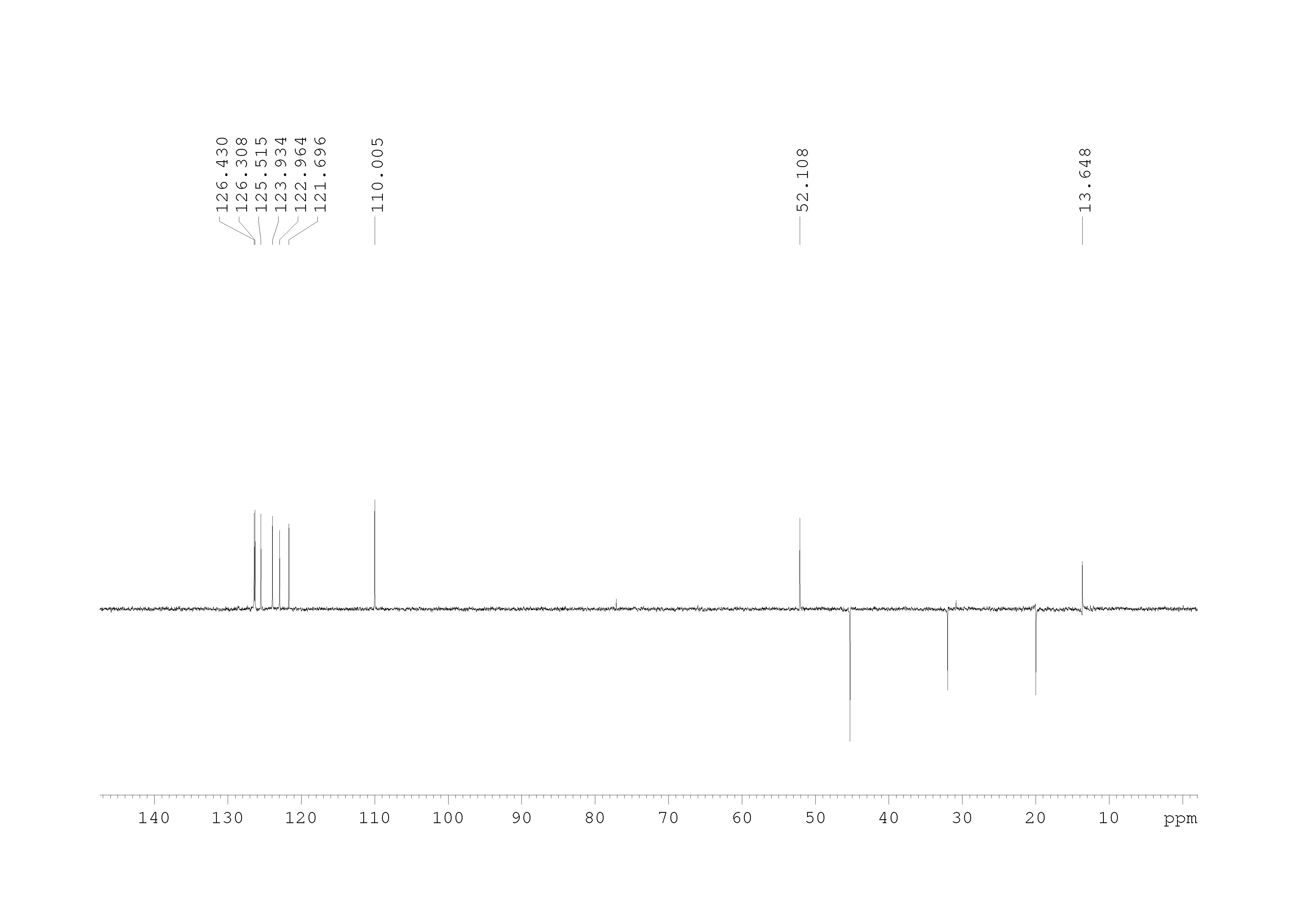


**Fig. S3.** DEPT spectrum in CDCl_3_ for N^N ligand.

 **Fig. S4.** ^1^H NMR of OncoIr3 in DMSO-d_6_.


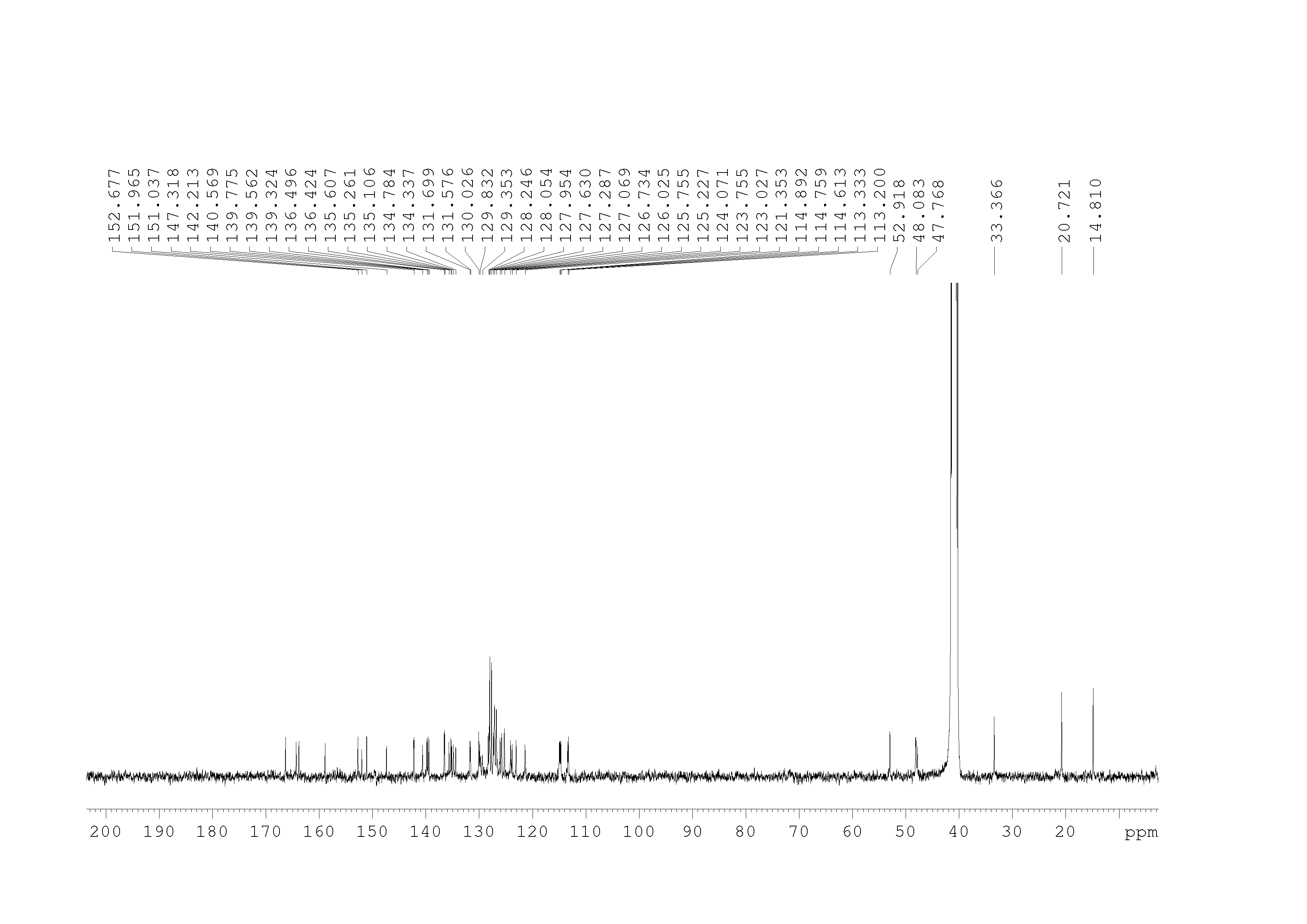


**Fig. S5.** ^13^C NMR of **OncoIr3** in DMSO-d_6_.

**Fig. S6.** Positive-ion ESI-MS of **OncoIr3**.

# Stability and purity studies

- 1. ***Stability assays***

The stability of the new complex **OncoIr3** was evaluated by UV/Vis spectrophotometry and NMR spectroscopy. For the stability in DMSO, the complex was dissolved in DMSO-d_6_ and the NMR spectrum was measured at room temperature at t=0 h and after 5 days. The stability was also checked in Roswell Park Memorial Institute (RPMI) culture medium by UV-Visible dissolving the complex in RPMI/DMSO (99:1) (10 μM).

- 1. ***RP-HPLC purity***

The purity of the Ir(III) complex was analyzed by using an RP-HPLC/MS TOF 6220 equipped with a double binary pump (model G1312A), degasser, autosampler (model G1329A), diode array detector (model G1315D), and mass detector in series (Agilent Technologies 1200). Chromatographic analyses were performed with a Brisa C18 column (150 mm x 4.6 mm, 5 µm particle size); Teknokroma, Macclesfield, UK. The mobile phase was a mixture of (A) H2O/HCOOH 0.1 % and (B) acetonitrile/HCOOH 0.1 %. The flow rate was 0.6 mL min^-1^ in a linear gradient. Chromatograms were recorded at λ=280 nm. The HPLC system was controlled by a ChemStation software (MASS HUNTER). The mass detector was an ion-trap spectrometer equipped with a dual-source electrospray APCI. Mass spectrometry data were acquired in the positive ionization mode. The ionization conditions were adjusted at 3508 ºC and 3 kV for capillary temperature and voltage, respectively.

| **Table S1.** HPLC method | | |
| --- | --- | --- |
| Time (min) | 0.1 % formic acid in dH_2_O | 0.1 % formic acid in CH_3_CN |
| 0-14 | 90 | 10 |
| 14-19.5 | 10 | 90 |
| 19.6-24 | 90 | 10 |

**Fig. S7.** HPLC chromatogram of **OncoIr3**.

**Fig. S8.** ^1^H-NMR spectra of **1** measured after dissolving immediately in DMSO-d_6_ (bottom) and after 5 days (top) at RT.


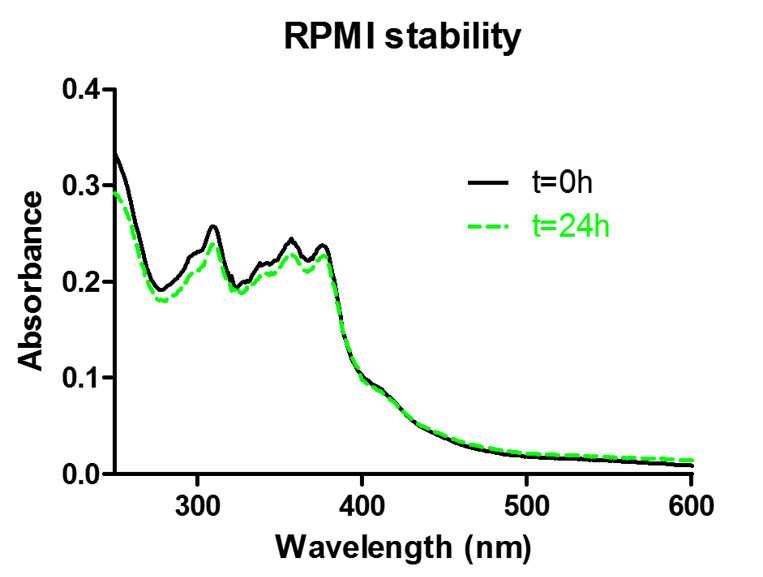


**Fig. S9.** Stability of **OncoIr3** in RPMI cell culture media after 24 h.

# Photophysical properties

UV/Vis spectroscopy was performed with a PerkinElmer Lambda 750S spectrometer with operating software. Emission spectra were obtained with a Horiba Jobin Yvon Fluorolog 3–22 modular spectrofluorometer with a 450 W xenon lamp. Measurements were performed in a right-angle configuration by using 10 mm quartz fluorescence cells for solutions at 25 ºC. Emission lifetimes (t) were measured by using an IBH FluoroHub TCSPC controller and a NanoLED pulse diode excitation source (t < 10 µs); the estimated uncertainty was ± 10 % or better. Emission quantum yields (Ф) were measured by using a Hamamatsu C11347 Absolute PL Quantum Yield Spectrometer; the estimated uncertainty was ± 5 % or better. Solutions of complex **1** were prepared in acetonitrile and water (1 % DMSO) at 10 μM. The change in the emission intensity was studied in a mixture of water and DMSO with different water fractions.

**Table S2.** Excitation (*λ*_ex_), emission (*λ*_em_) wavelengths, emission lifetimes (*τ*_em_), and quantum yields (*Φ*_em_) of **1** in different solvents.

| **Solvent** | **λ_exc_, nm^a^** | **λ_em_, nm** | **τ_em_, ns^b^** | **τ_em_, ns^c^** | **Փ_em_,%^b^** | **Փ_em_,%^c^** |
| --- | --- | --- | --- | --- | --- | --- |
| Acetonitrile | 372 | 665 | 223 (100%) | 393 (96%)  744 (4%) | 3.9 | 6.9 |
| Water (1% DMSO) | 376 | 661 | 181 (13%)  413 (87%) | 370 (89%)  1237 (11%) | 6.0 | 6.1 |

^[a]^ λ_exc_ maxima. ^[b]^ Emission lifetime and emission quantum yields measured in aerated solution. ^[c]^ degassed by bubbling 20 min under argon

**Fig. S10.** UV/Vis absorption and emission spectra of **OncoIr3** in acetonitrile (---, black) and water (˗˗, red) at room temperature. The excitation wavelength was 405 nm.

**Fig. S11.** Emission spectra of **OncoIr3** (2-benzothiazol-y group) and the complex with the 2-pyridin-yl group described previously for some of us [2]. Medium: Water (1 % DMSO).

**Fig. S12.** Emission spectra of **OncoIr3** in water at different excitation wavelengths.

# *In vitro* biological evaluation

- 1. **Cell uptake and co-localization studies**

| A2780 | HeLa |
| --- | --- |
| **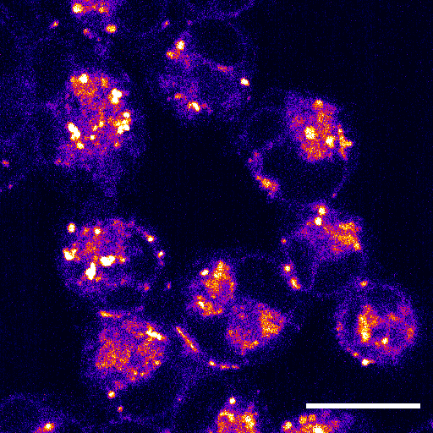** | **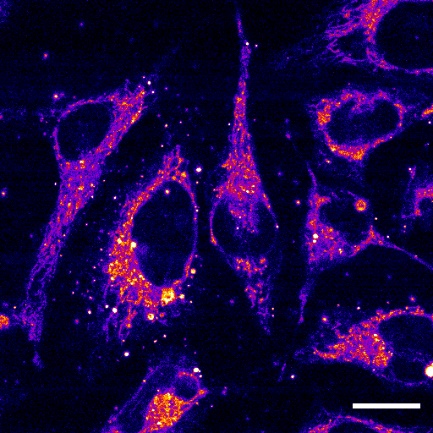** |

**Fig. S13.** Cellular uptake of **OncoIr3** in A2780 and HeLa cells. Single confocal planes of cancer cells incubated with the compound (5 µM; 30 min) at 37ºC using λ_exc/em_ = 405/630 nm. Confocal images are color-coded using Fire lookup table from FIJI software. Scale bar: 20 µm (left; A2780); 15 µm (right; HeLa).

.

.

| 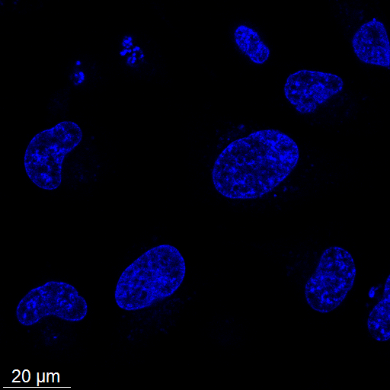 | 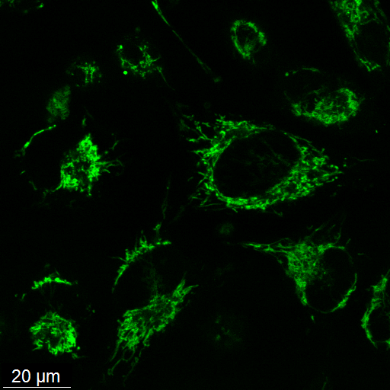 | 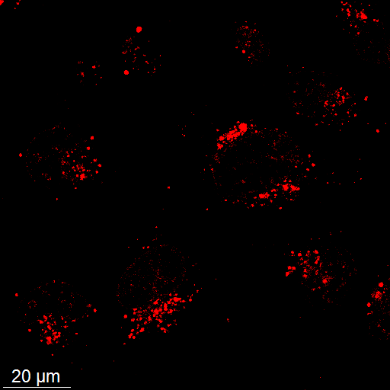 | 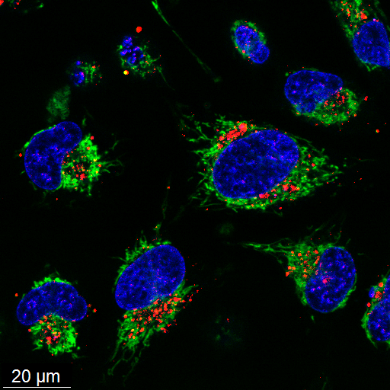 |
| --- | --- | --- | --- |
| DAPI | MitoTracker Green | OncoIr3 | Merge |

**Fig. S14.** Colocalization assays in HeLa cells after MitotrackerGreen staining (MTG; 0.1 µM; 30 min) and **OncoIr3** (5 µM; 30 min) at 37ºC. Pearson and Manders correlation coefficients were calculated as average of at least 15 Z-stack images using the JACoP plugin in FIJI software.

- 1. **Antiproliferative activity**

| Table S3. IC_50_ values [µM] of OncoIr3 and cisplatin after 48 h. | | | | | | | | |
| --- | --- | --- | --- | --- | --- | --- | --- | --- |
|  | **A2780** | **A2780cis (RF^a^)** | **HeLa** | **MDA-MB-231** | | **CHO** | **BGM** | **SF^b^** |
| OncoIr3 | 0.19 ± 0.02 | 0.208 ± 0.01 (1.1) | 0.4 ± 0.1 | | 0.7 ± 0.2 | 2.6 ± 0.5 | >20 | 13.7 |
| Cisplatin | 2.4 ± 0.2 | 27 ± 4 (11.3) | 35 ± 3 | | 26 ± 4 | 8.6 ± 0.9 | 6 ± 1 | 3.6 |
| ^a^Resistance factor: IC_50_ A2780cis/IC_50_ A2780; ^b^selectivity factor: IC_50_ CHO /IC_50_ A2780 | | | | | | | | |

| Table S4. IC_50_ values [µM] of OncoIr3 after 3 h and 24 h. | | | | |
| --- | --- | --- | --- | --- |
|  | **A2780** | **A2780cis (RF^a^)** | **HeLa** | **MDA-MB-231** |
| 3 h | 5.8 ± 0.6 | 6.5 ± 0.9 (1.1) | 8.8 ± 0.3 | 9.6 ± 0.4 |
| 24 h | 0.91 ± 0.07 | 1.0 ± 0.1 (1.1) | 1.8 ± 0.3 | 2.4 ± 0.3 |
| ^a^Resistance factor: IC_50_ A2780cis/IC_50_ A2780 | | | |  |

|  | **Control** | **OncoIr3** | **Cisplatin** |
| --- | --- | --- | --- |
| A2780 | 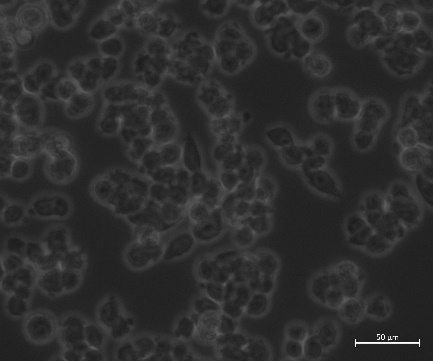 | 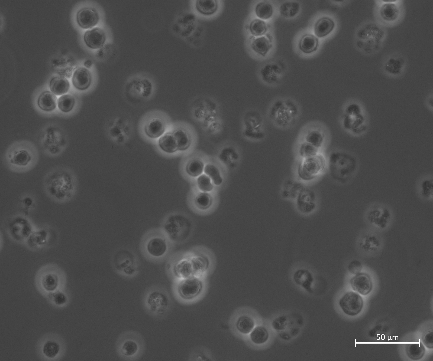 | 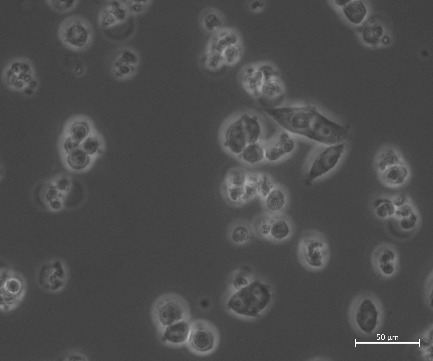 |
| HeLa | 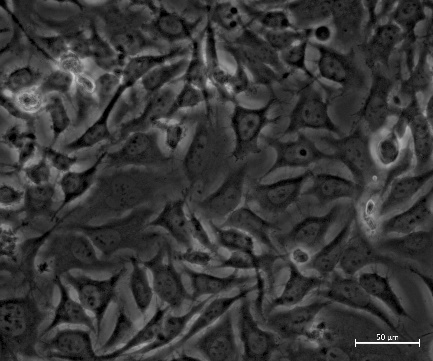 | **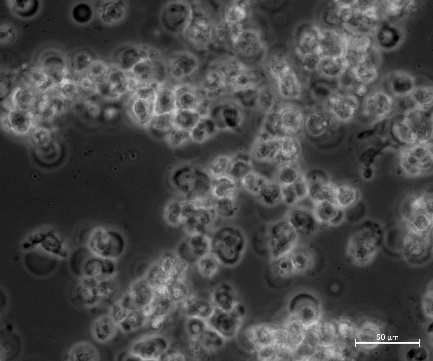** | 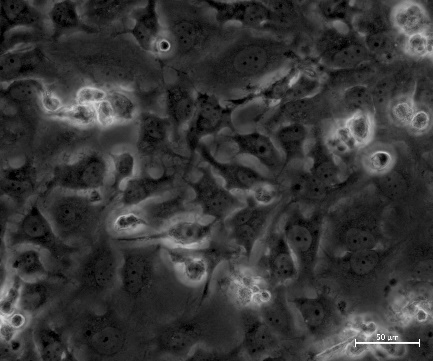 |

**Fig.** **S15.** Microscopy imaging of A2780 and HeLa cells after 24 h treatment with **OncoIr3** (5 µM) or cisplatin (close to IC_50_ concentration) visualized under phase contrast Zeiss Axio Observer 7 microscope. Scale bar = 50 µm.


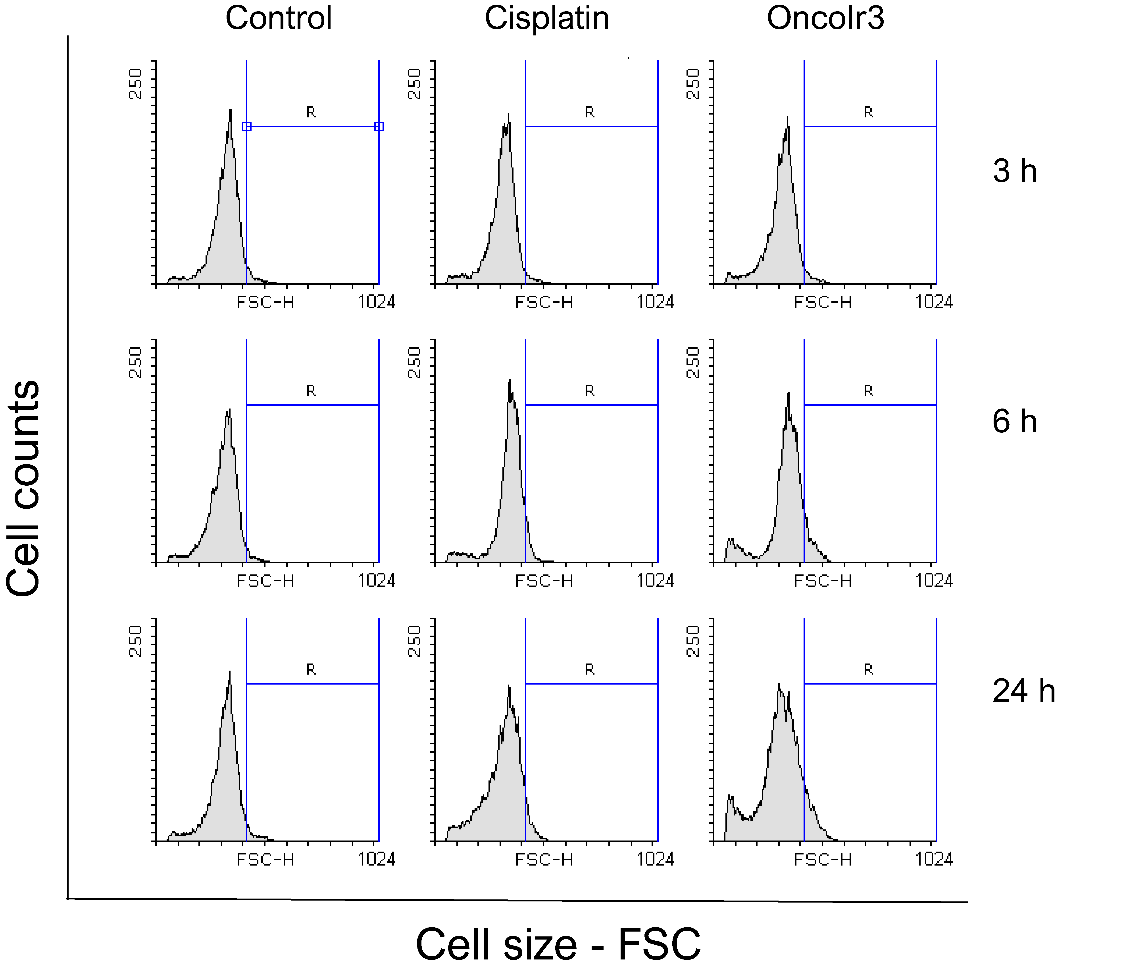


**Fig. S16**. Cell size changes of A2780 cells detected by flow cytometry in FSC channel upon treatment with cisplatin or **OncoIr3** (5 µM). Gated region (R) depicted as reference for high cell enlargement.


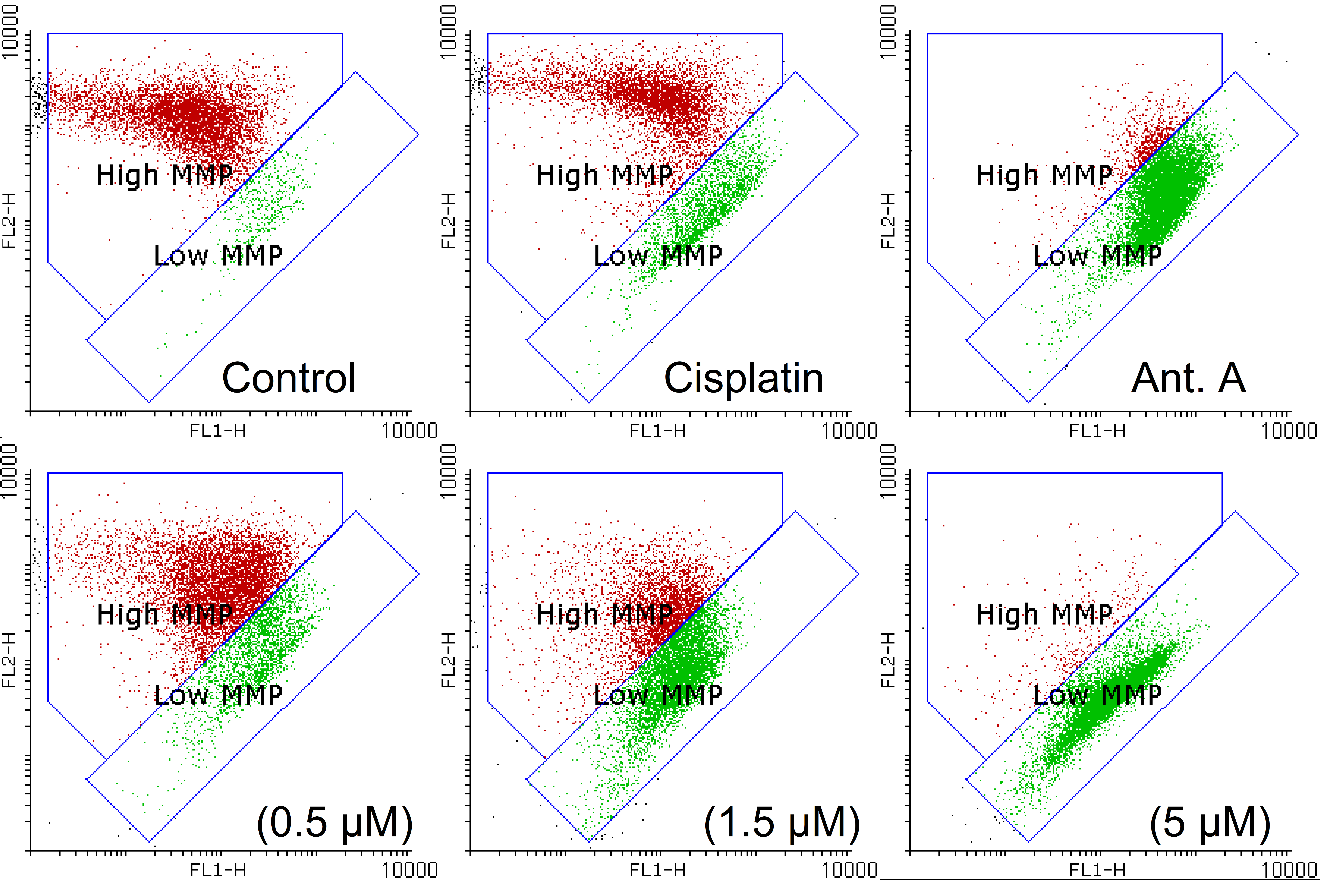


**Fig. S17.** Flow cytometry dot plots of A2780 cells treated for 24 h with cisplatin (5 µM), antimycin A (Ant. A; 50 µM) or **OncoIr3**. Mitochondrial membrane potential (MMP) changes detected as green JC‑1 dye monomers (low MMP) or red aggregates (high MMP) in FL1 and FL2 channels.

- 1. **ROS determination**

A2780 cells were seeded in 96‑well plates (2·10^5^ cells/well) for 24 h. Cells were then washed with PBS and incubated with 10 μM DHE for 30 min at 37 ºC avoiding direct light. The staining solution was removed, and the compounds diluted in cell medium without phenol red were added at appropriate concentrations for 3 or 6 h. Menadione 100 µM was used as a positive control. Cells were analyzed by flow cytometry (Fortessa X-20; 5·10^3^ events/sample) using Ex_488_/Em_590_ parameters to obtain fluorescence intensities that were normalized to untreated controls to calculate relative ROS levels. Experiments were performed in triplicate with n=2 wells per replicate.

**Fig. S18**. Relative ROS levels in A2780 cells measured with dihydroethidium (DHE; 10 µM for 30 min) after treatment with Menadione (50 µM) or **OncoIr3** at indicated concentrations. Statistical significance untreated control vs. treatment *p<0.05, **p<0.01, ***p<0.001 from multiple unpaired t-test.

- 1. **Membrane integrity test by flow cytometry**

**
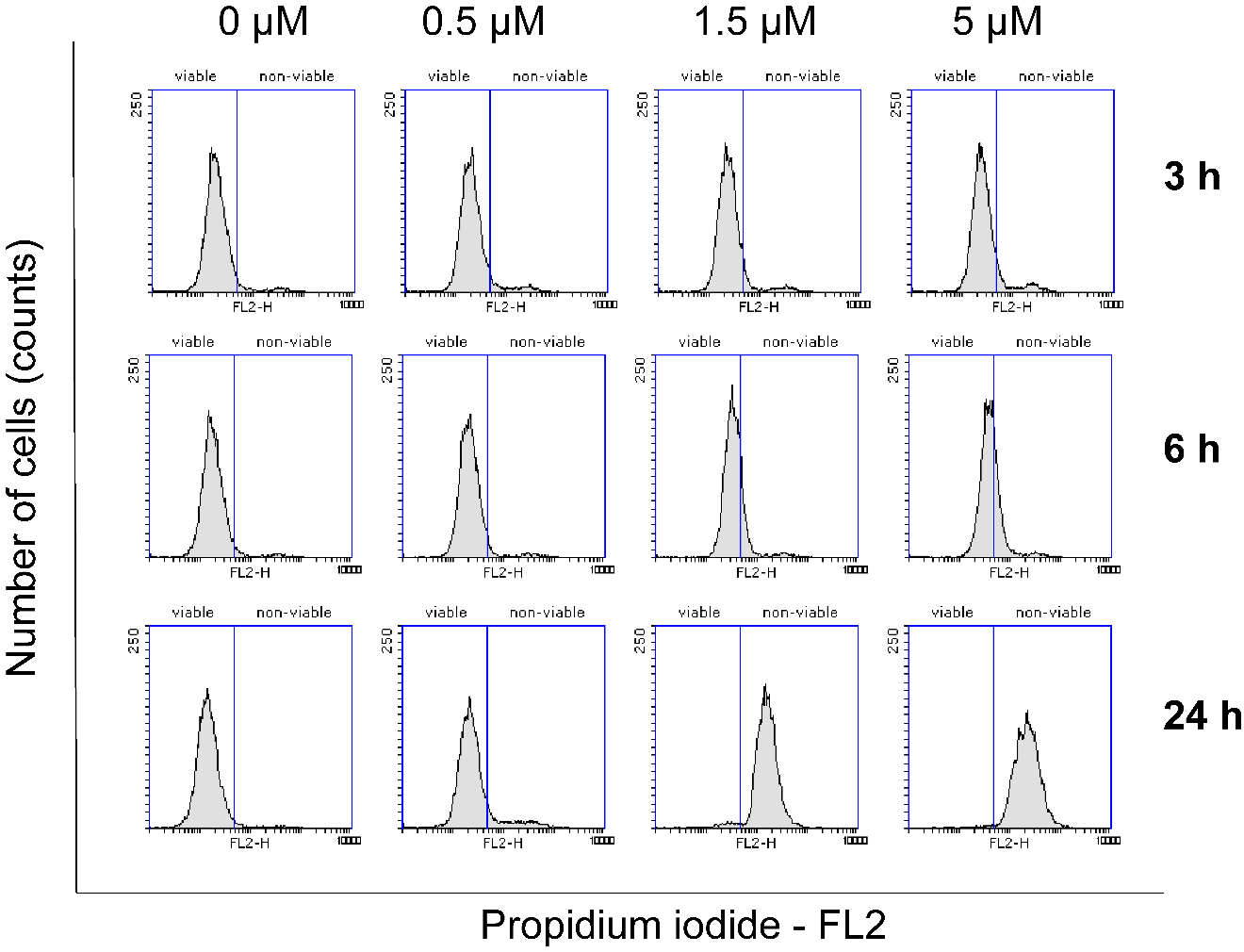
Fig. S19.** Membrane integrity assessment of A2780 cells after **OncoIr3** treatment determined by intracellular propidium iodide fluorescence detection in FL2 channel. Histograms were gated as viable membrane (FL2-) and non-viable membrane (FL2+).

- 1. **Cell death studies**


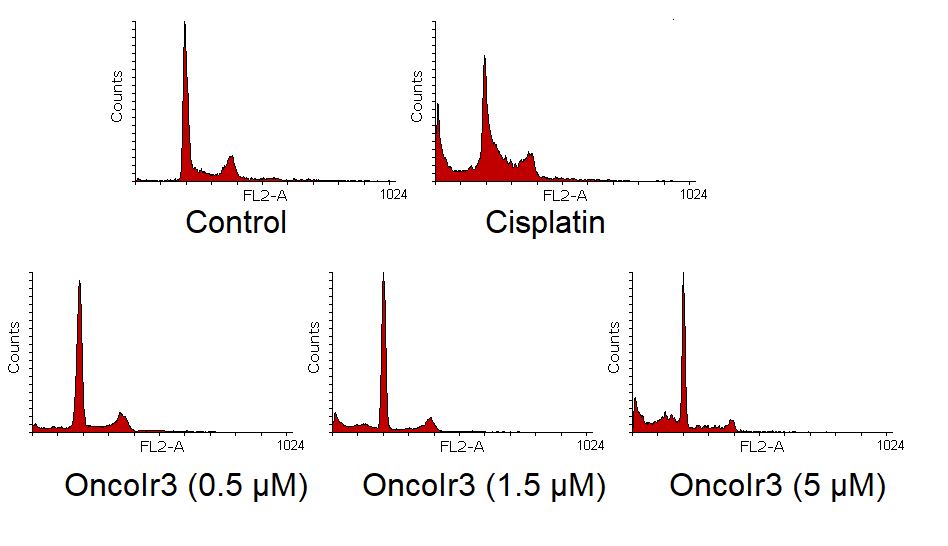


**Fig. S20**. Representative cell cycle histogram analysis of A2780 cells 24 h treatment with **OncoIr3** or cisplatin (5 μM) detected by propidium iodide staining in FL2 channel.

**
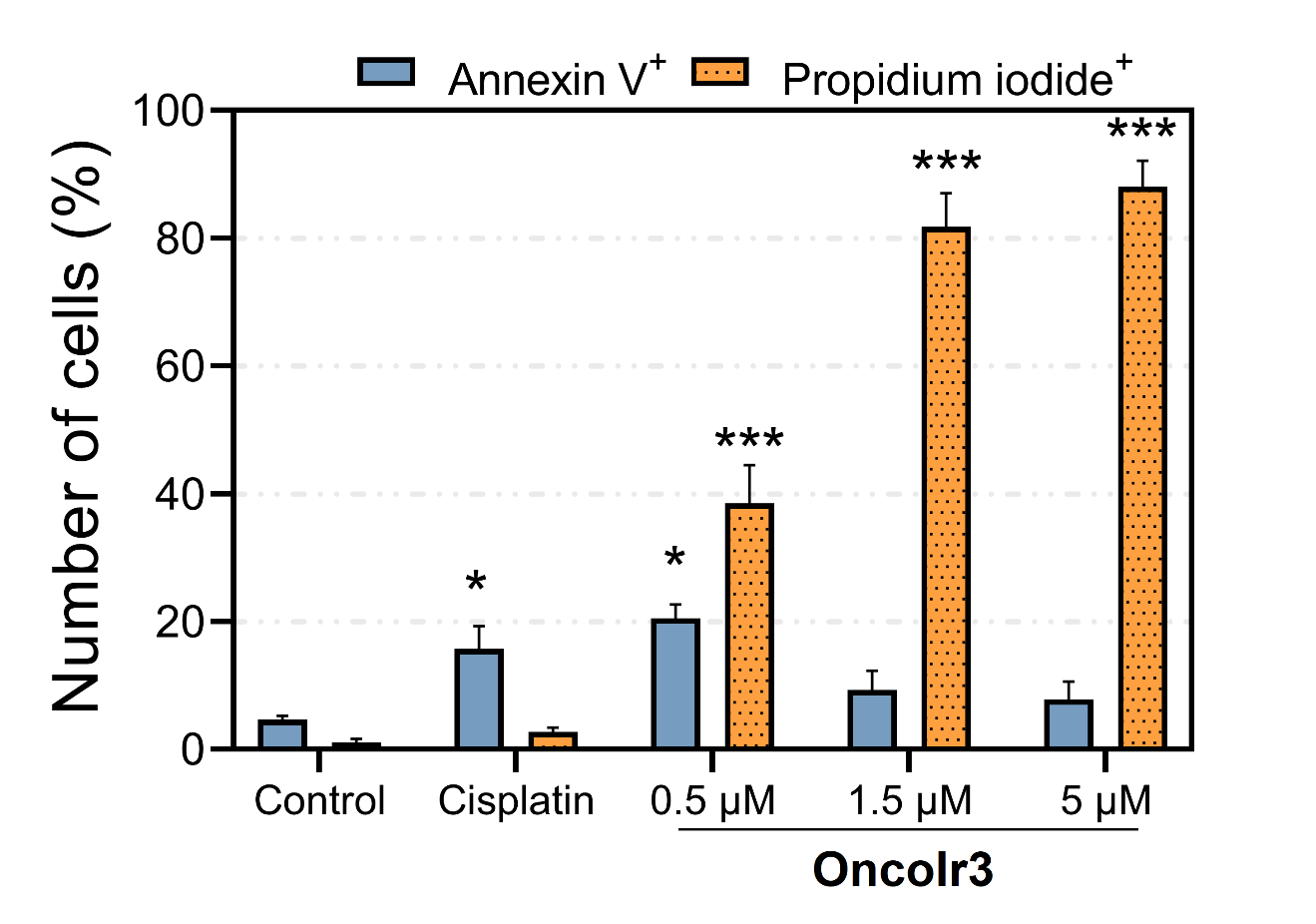
**

**Fig. S21**. Cell death induction in A2780 cells after 24 h treatment with OncoIr3 or cisplatin (5 μM) determined by Annexin V-FITC/Propidium iodide double staining. Data expressed as mean ± SD from three independent experiments (*p < 0.05, **p < 0.01, ***p < 0.001; One-way Anova test).


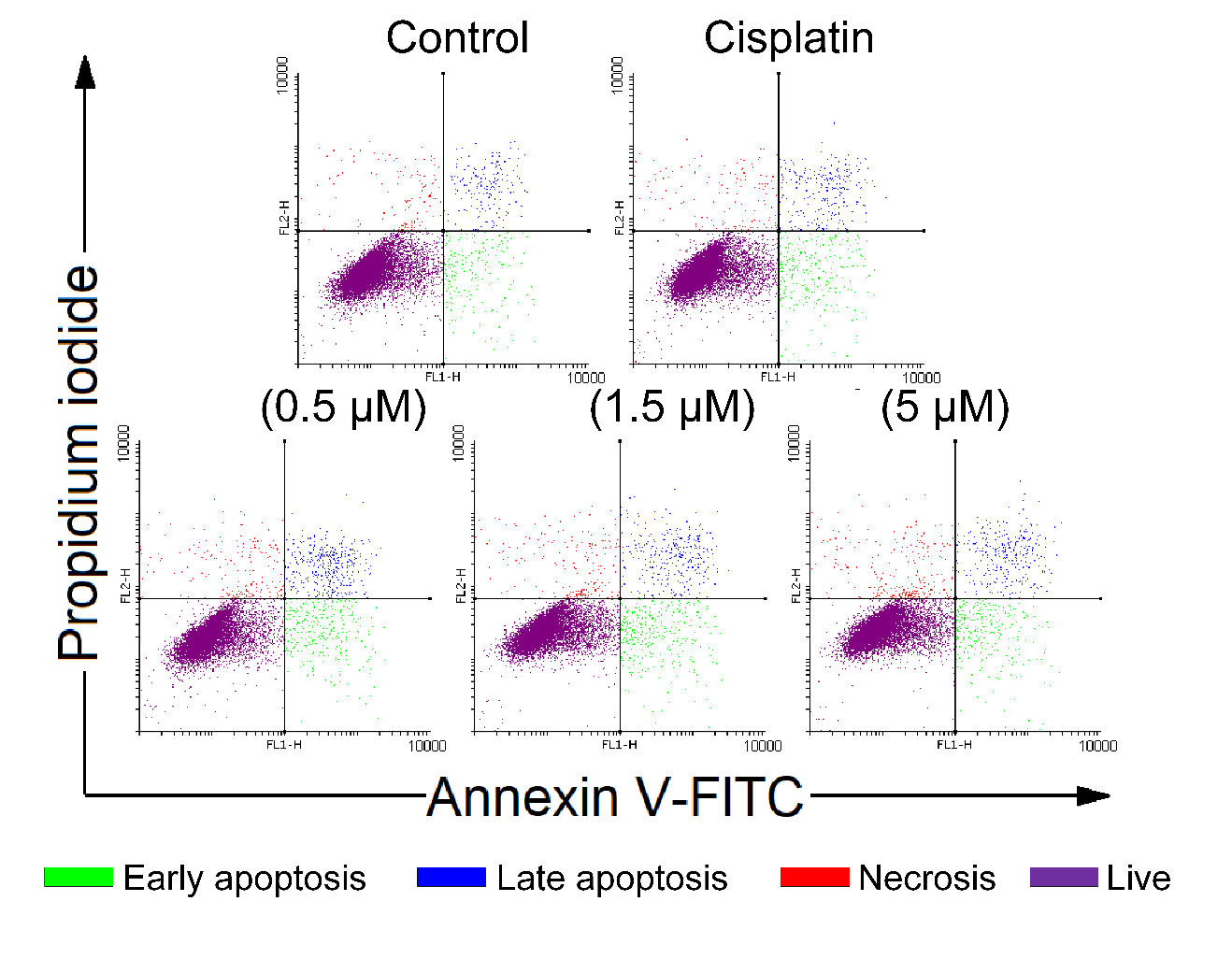


**Fig. S22**. Representative dot plots analysis of A2780 cells 3 h treatment with **OncoIr3** or cisplatin (5 μM) following double-staining AnnexinV-FITC(FL1)/Propidium iodide(FL2) method.


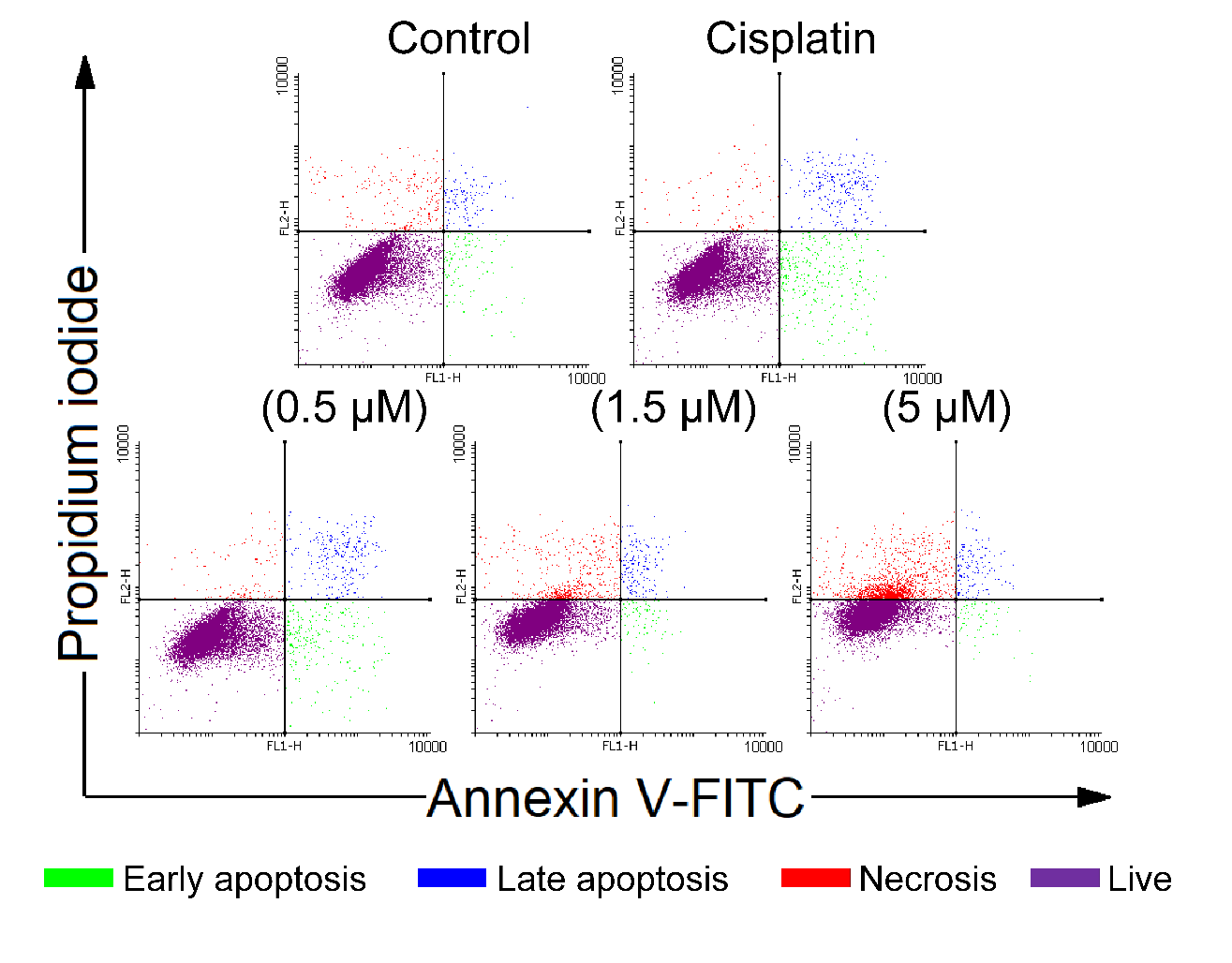


**Fig. S23**. Representative dot plots analysis of A2780 cells 6 h treatment with **OncoIr3** or cisplatin (5 μM) following double-staining Annexin V-FITC(FL1)/Propidium iodide(FL2) method.


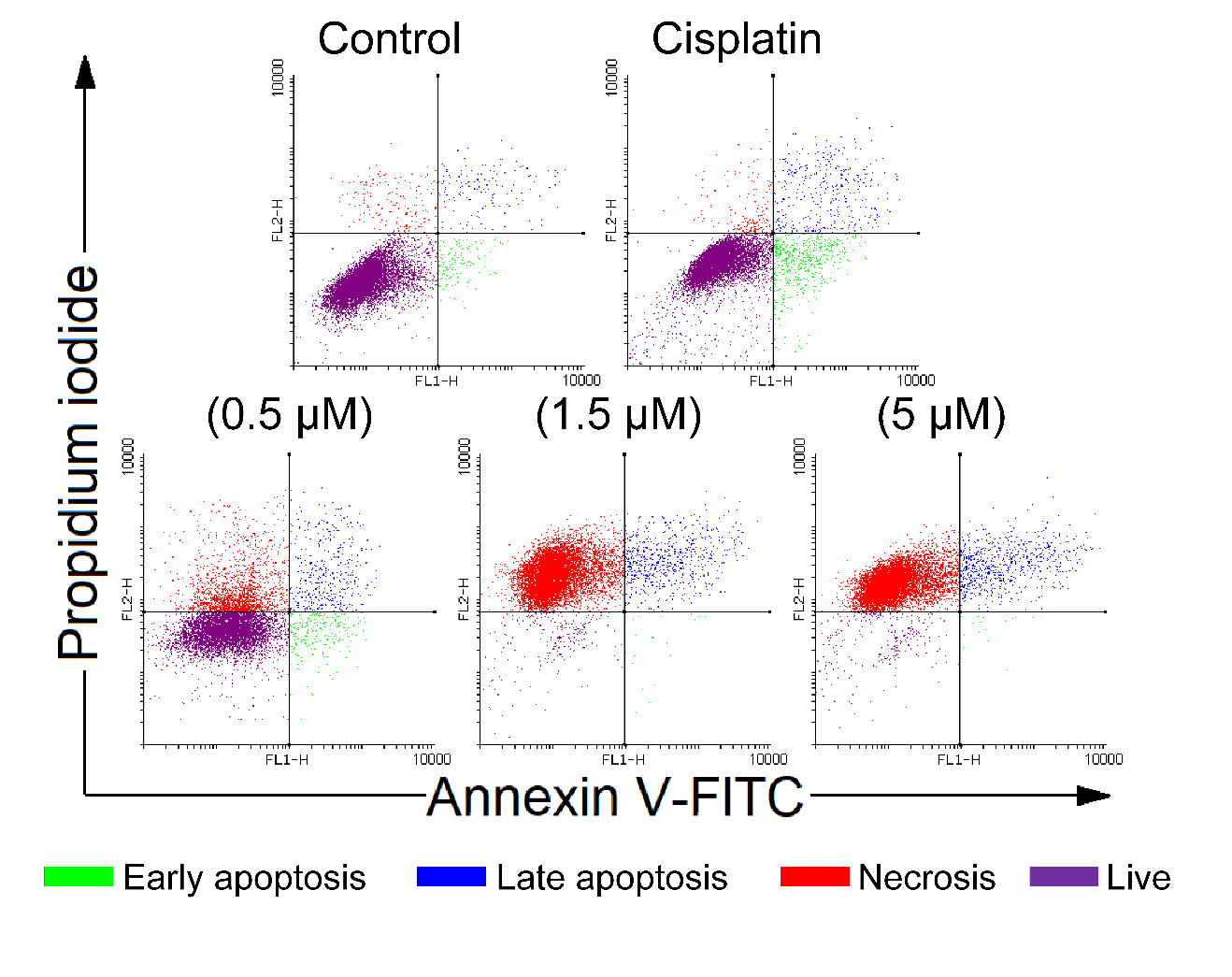


**Fig. S24**. Representative dot plots analysis of A2780 cells 24 h treatment with **OncoIr3** or cisplatin (5 μM) following double-staining Annexin V-FITC(FL1)/Propidium iodide(FL2) method.

| **a** |  |
| --- | --- |
| **b** |  |

**Fig. S25**. Growth inhibition of **OncoIr3** in A2780 cells after pretreatment with various inhibitors. (**a**) Pharmacological inhibition of apoptosis and paraptosis pathways using NSCI (5 μM), pifithrin-alpha (10 μM), Cycloheximide (CHX, 100 μM). (b) Pharmacological inhibitions of necrosis-related pathways using leupeptin (100 μM), Necrostatin-1 (60 μM). Cells were pre-incubated for 1 h with inhibitors and then treated with **OncoIr3** for 24 h.

- 1. **Wound Healing assays.**

The wound healing ability of the iridium complex was evaluated in A2780 cancer cells. Briefly, well‑defined wounds were created in the cell layer using Ibidi™ cell culture inserts. Then 6·10^4^ cells were in complete growth medium and allowed to reach confluence in and humidified incubator. Cell inserts were then removed using sterile forceps to create the gap and the cells were washed 3 times to remove floating or dead cells. Next, either 0.4 % DMSO or the iridium compound (0.5 µM) was added to the cells. Images were recorded using phase contrast microscopy (NIKON Eclipse TE 2000U microscope) every 24 h. Images were processed with Fiji software and the percentage of wound closure was obtained using the next the equation:

$$\% wound closure=\frac{A_{t=0h}-A_{t=\Delta h}}{A_{t=0h}}$$

Where *A* is the area of the wound at a given time (*t).* The experiment was performed in triplicate.

*
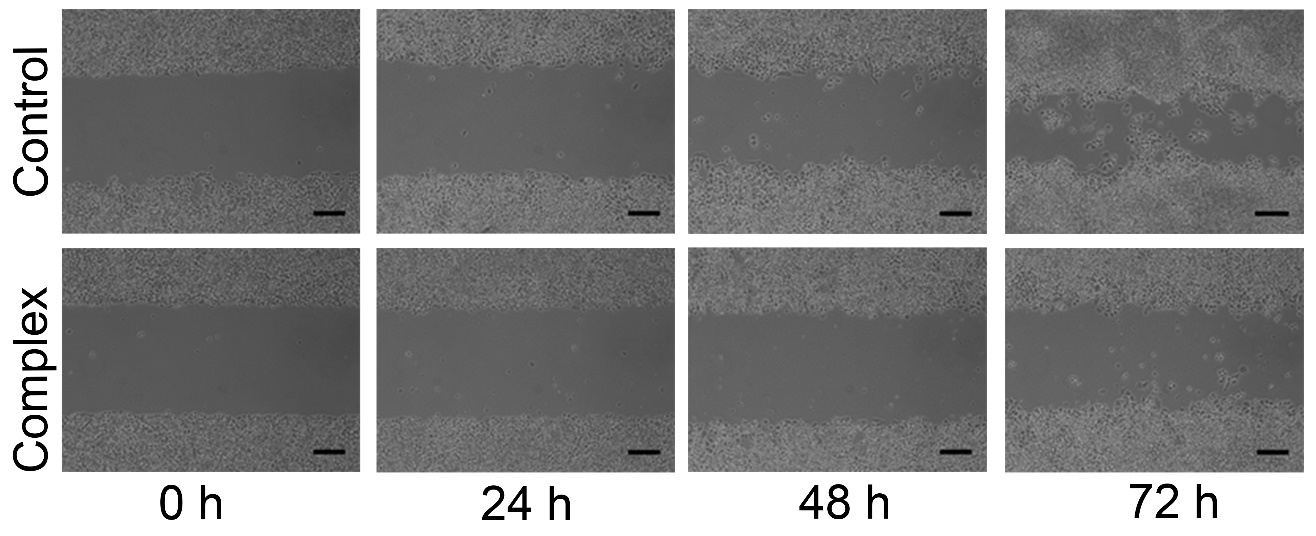
*******

**Fig. S26**. Cell migration tested by wound-healing assay. A2780 cells were treated with OncoIr3 (0.5 μM). Scale bar: 100 μm. Data expressed as average ± SD from three replicates. Statistical significance control *vs.* treatment from unpaired t-test (**p < 0.01).

# *In vivo* biological evaluation

**Table S5.** *In vivo* measurements of tumor size

|  | C (µM) | n | Tumor Area (µm^2^) | S.D | Reduction (%) | *p* value *vs* control |
| --- | --- | --- | --- | --- | --- | --- |
| Control |  | 39 | 14169.02 | 2613.06 | 0.00 |  |
| CDDP | 100 | 24 | 8149.27 | 1072.96 | -42.49 | <0.00001 |
| DMSO (0.4 %) |  | 20 | 13622.75 | 1287.38 | 0.00 | 0.099197^a^ |
| OncoIr3 | 0.1 | 47 | 12193.85 | 2703.41 | -10.49 | <0.00001 |
|  | 1 | 21 | 9517.95 | 1558.20 | -30.13 | <0.00001 |
|  | 10 | 20 | 8794.06 | 1461.13 | -35.45 | <0.00001 |
|  | 100 | 42 | 8033.38 | 2067.55 | -41.03 | <0.00001 |

^a^ Control *vs* DMSO control


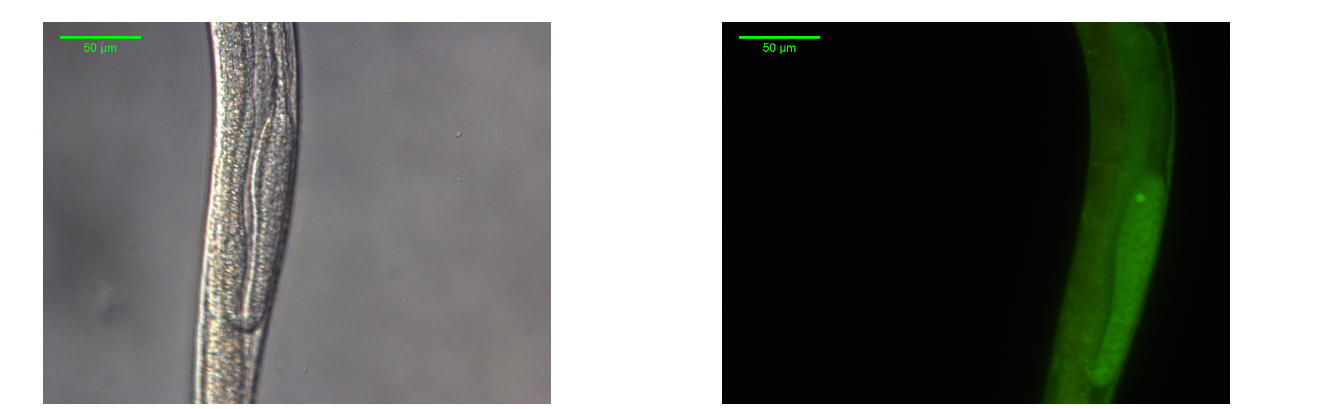


**Fig. S27.** Tumoral gonads of JK1466 animals treated with 100 μM of **OncoIr3** visualized with DIC (left) and stained with AO (right). Scale bar: 50 μm.

- 1. ***In vivo* ROS generation**

ROS generation was measured with DHE (dihydroethidium) following the published protocol of Dues and coauthors [5] with slight modifications. Briefly, synchronized wild-type L1 larvae were treated with **OncoIr3** (100 µM), cisplatin (100 µM), DMSO (0.4 %) or paraquat (methyl viologen 200 µM) for 72 hours at 20 ºC. Then the animals were collected and washed three times with PBS buffer and stained with 500 µL of a DHE solution (30 µM in PBS) for one hour at 37 ºC under orbital shaking. Following the staining procedure, the animals were visualized under fluorescent light using the N2.1 filter cube and the 40x lens. The analysis of the images was performed with ImageJ software using only the red channel. Two independent assays were performed with n ≥ 10 and the statistical significance was estimated by ANOVA test.

**
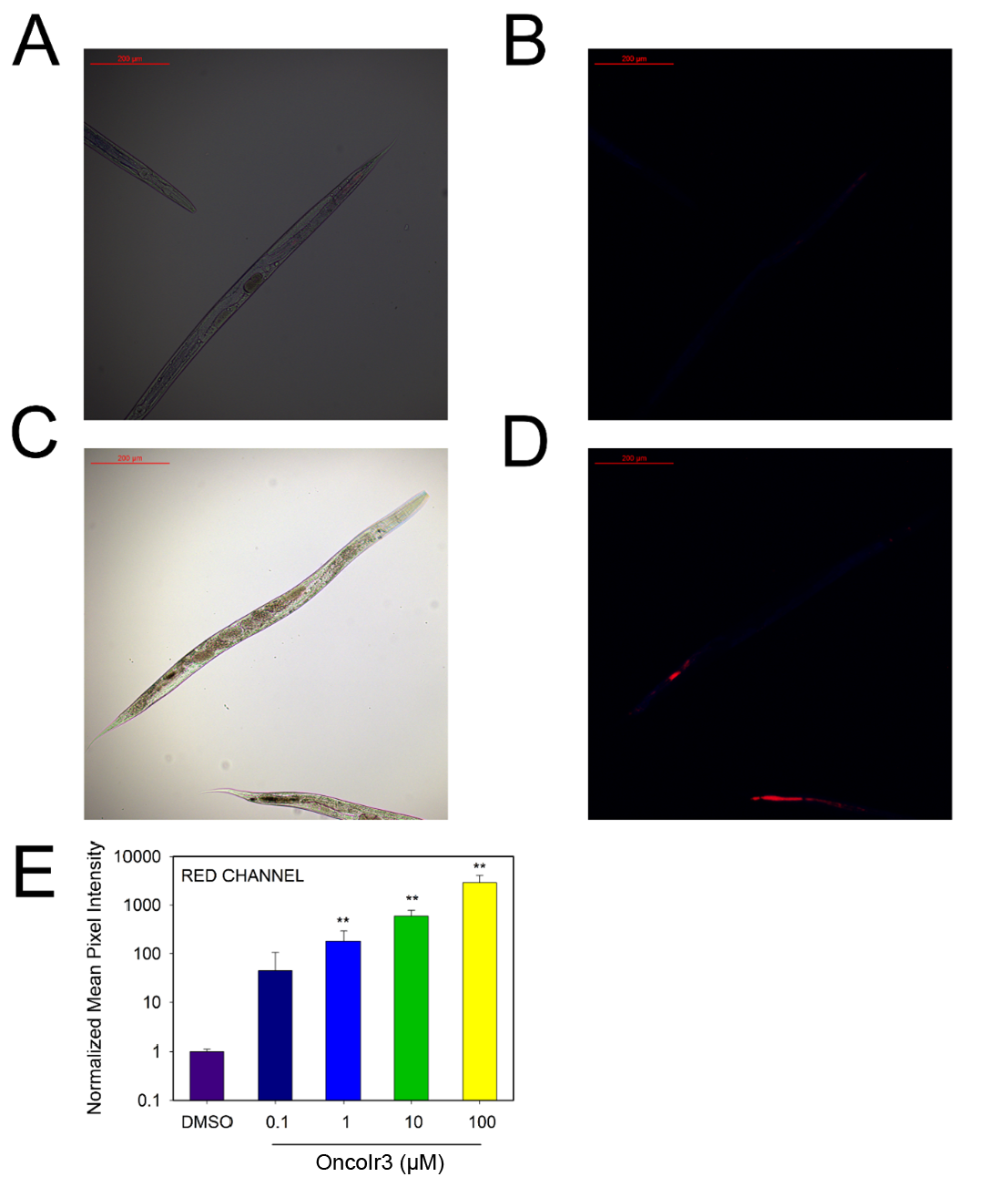
**

**Fig. S28. Accumulation of OncoIr3 *in vivo*.** **A-E** Representative images of N2 animals exposed to the iridium complex. **A** Brightfield at 10 µM. **B** Fluorescence image at 10 µM. **C** Brightfield at 100 µM. **D** Fluorescence image at 100 µM. **E** Quantification of the fluorescence intensity inside the animals, data are represented as mean ± S.D, n = 15 per condition, two independent experiments were performed, **significantly at *p* ≤ 0.05 by ANOVA test. Scale bars 200 µm.

**
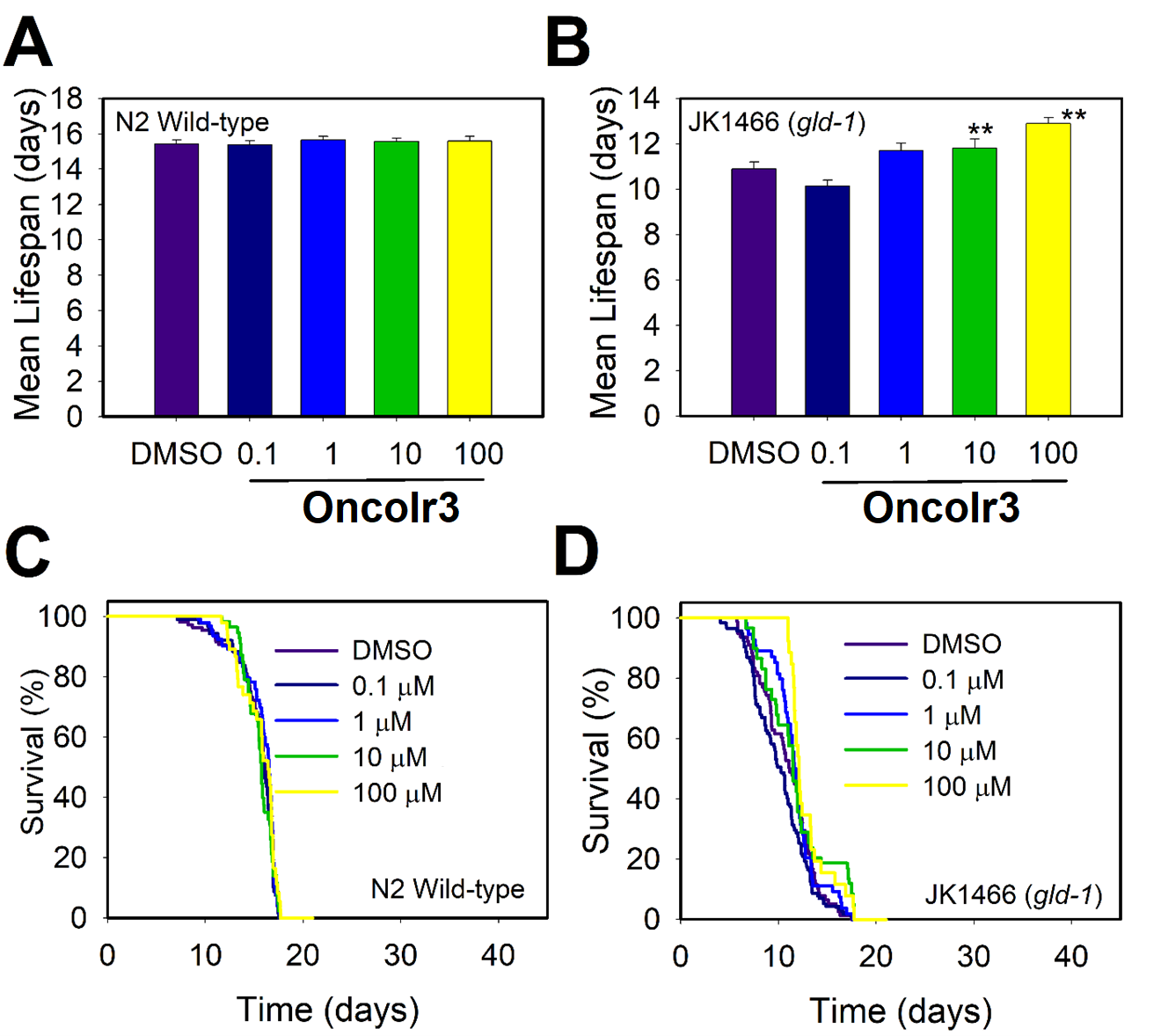
**

**Fig. S29. Toxicity effects of the iridium complex**. **A** Mean lifespan of N2 animals treated with different concentrations of the iridium complex, Data is represented as mean lifespan ± S.E, **significantly at *p* ≤ 0.05 by Log Rank test. **B** Mean lifespan of JK1466 strain treated with different concentrations of the iridium complex. Data are represented as mean lifespan ± S.E, **significantly at *p* ≤ 0.05 by Log Rank test. **C-D** Survival curves for the wild-type strain **C** and the tumoral strain **D**

**
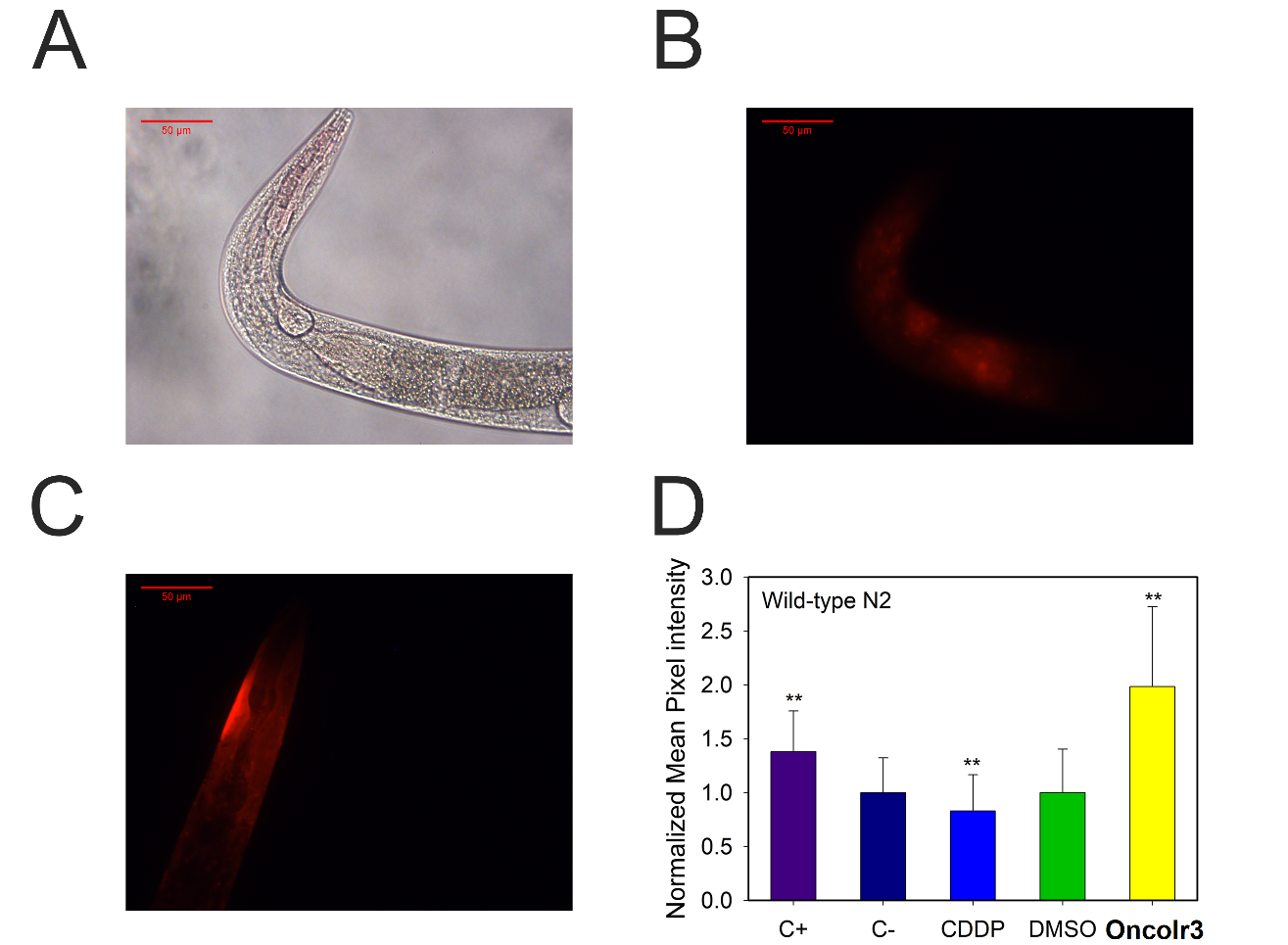
**

**Figure S30. *In vivo* effects of OncoIr3 in superoxide generation and in non-apoptotic cell death. A-C** Representative images of wild-type *C. elegans* stained with DHE. **A-B** Positive control (200 μM paraquat) worm in brightfield **(A)** or in fluorescence channel (**B**) and **OncoIr3**-treated with 100 μM (**C**). Quantification of superoxide levels presented as mean ± SD, n = 15 per condition, two independent experiments were performed, **significantly at *p* ≤ 0.05 by ANOVA test.

**Table S6.** *C. elegans* survival analysis

| Strain |  | C (µM) | n | Mean lifespan (days) | S.E | 95 C.I^a^ | Change (%) | *p* value *vs* control |
| --- | --- | --- | --- | --- | --- | --- | --- | --- |
|  |  |  |  |  |  |  |  |  |
| N2 | **OncoIr3** | 0 | 109 | 15.42 | 0.22 | 14.99 ~ 15.85 | 0.0000 |  |
|  |  | 0.1 | 90 | 15.38 | 0.22 | 14.95 ~ 15.80 | -0.2594 | 0.1254 |
|  |  | 1 | 95 | 15.65 | 0.21 | 15.24 ~ 16.06 | 1.4916 | 0.9446 |
|  |  | 10 | 97 | 15.56 | 0.2 | 15.17 ~ 15.95 | 0.9079 | 0.3134 |
|  |  | 100 | 88 | 15.58 | 0.28 | 15.04 ~ 16.12 | 1.0376 | 0.8895 |
| Jk1466  (*gld-1(-)*) | **OncoIr3** | 0 | 80 | 10.89 | 0.32 | 10.27 ~ 11.51 | 0.0000 |  |
|  |  | 0.1 | 115 | 10.14 | 0.27 | 9.61 ~ 10.66 | -6.8306 | 0.1614 |
|  |  | 1 | 94 | 11.69 | 0.33 | 11.05 ~ 12.34 | 7.2860 | 0.3807 |
|  |  | 10 | 99 | 11.8 | 0.42 | 10.97 ~ 12.64 | 8.2878 | 0.0476 |
|  |  | 100 | 82 | 12.89 | 0.27 | 12.36 ~ 13.43 | 18.2149 | 0.0052 |

^a^ Confidence interval 95 %

- 1. ***C. elegans lethality and sublethality assays***

Further evaluation of the chemical toxicity of **OncoIr3** was performed increasing the compound dose for the animals. L1 larvae were exposed to 100 µM, 500 µM and 2 mM of **OncoIr3** maintaining the DMSO concentration fixed at 0.4 % (v/v) in S basal medium supplemented with *E. coli* OP50 for 48 hours at 20 ºC. Samples were taken and the nematodes development and survival rates were scored. For the lethality assay individuals exposed to either **OncoIr3** or DMSO were transferred to a clean NGM plates, visualized and recorded under a stereomicroscope (OPTIKA S.r.l., Italy) equipped with an Optika H series camera (OPTIKA S.r.l., Italy). Animals that failed to move when irradiated with the macroscope light were scored as dead. *C. elegans* body length and development were studied using bright field microscopy, images of animals treated with the compound or DMSO (control) were taken and analyzed using imageJ software. Body length is measured from the tip of the nose to the tail of each animal.


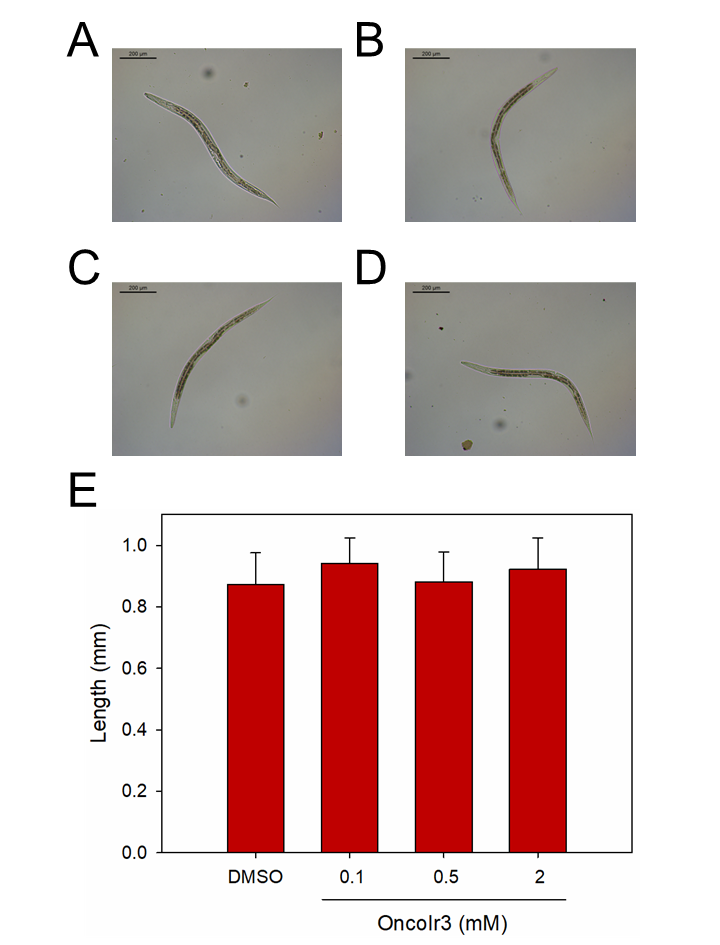


**Figure S31. OncoIr3 effects on *C. elegans* size and development. A-D** Representative brightfield images of wild-type *C. elegans* exposed to different concentrations of the compound. (**A)** Control (0.4 % DMSO) (**B**) **OncoIr3** 100 μM (**C**). **OncoIr3** 500 μM (**D**) **OncoIr3** 2 mM. Scale bar= 200 µm (**E**) Quantification of animals’ body length presented as mean ± SD, n = 35 per condition, two independent experiments were performed.

- 1. ***C. elegans locomotion assay***

The animal’s motility was studied by analyzing recorded videos of *C. elegans* exposed to **OncoIr3** using the plugin for ImageJ wrMTrckr available in <http://www.phage.dk/plugins/wrmtrck.html>. The animal locomotion was measured following the guide “*C. elegans* motility analysis in ImageJ-A practical approach” also available in https://www.phage.dk/plugins/download/wrMTrck.pdf [6].


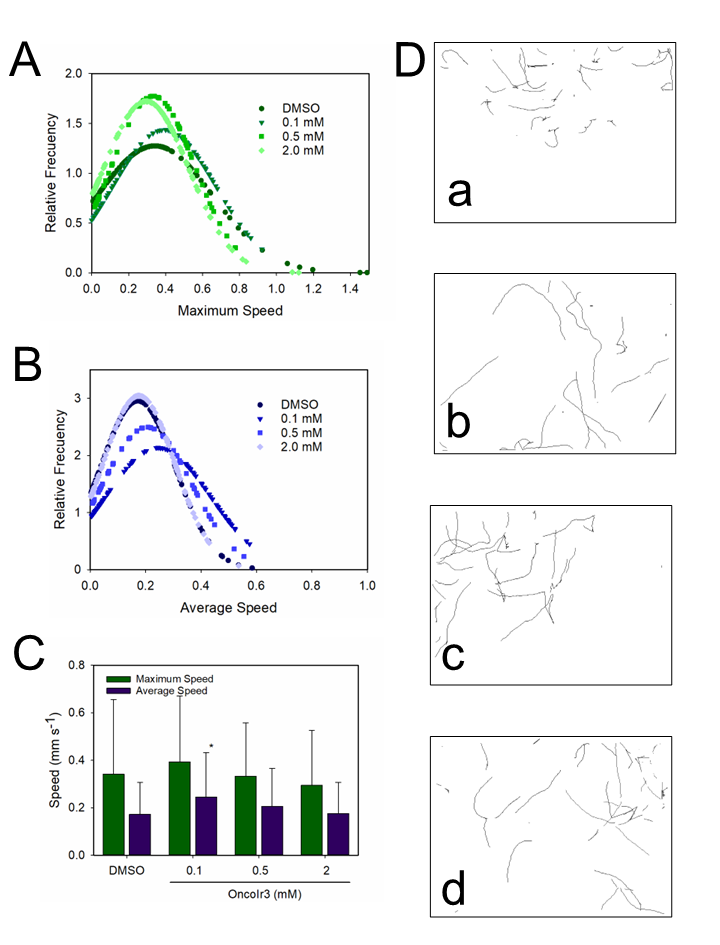


**Figure S32. OncoIr3 effects on *C. elegans* motility.** (**A**) Maximum speed normal distribution for animals exposed to increasing concentrations of **OncoIr3**. (**B**) Average speed under the same conditions. (**C**) Quantification of animals’ speed presented as mean ± SD, n = 35 per condition, two independent experiments were performed, **significantly at *p* ≤ 0.05 by ANOVA test. (**D**) Representative motility patterns of wild-type *C. elegans* exposed to different concentrations of the compound. (**a)** Control (0.4 % DMSO) (**b**) **OncoIr3** 100 μM (**c**). **OncoIr3** 500 μM (**d**) **OncoIr3** 2 mM.

**Supplementary Video 1: *C. elegans* locomotion patterns when exposed to different concentrations of OncoIr3**. Above, raw videos for each condition are shown, while below, the transformed versions of the same videos for software analysis are shown.

**SI References**

[1] Yellol, J. *et al.* Novel C,N-Cyclometalated Benzimidazole Ruthenium(II) and Iridium(III) Complexes as Antitumor and Antiangiogenic Agents: A Structure–Activity Relationship Study. *J. Med. Chem.* **58**, 7310–7327 (2015).

[2] Yellol, J. *et al.* Highly potent extranuclear-targeted luminescent iridium(iii) antitumor agents containing benzimidazole-based ligands with a handle for functionalization. *Chem. Commun.* **52**, 14165–14168 (2016).

[3] Cao, J.-J. *et al.* Anticancer Cyclometalated Iridium(III) Complexes with Planar Ligands: Mitochondrial DNA Damage and Metabolism Disturbance. *J. Med. Chem.* **62**, 3311–3322 (2019).

[4] Pracharova, J. *et al.* Exploring the Effect of Polypyridyl Ligands on the Anticancer Activity of Phosphorescent Iridium(III) Complexes: From Proteosynthesis Inhibitors to Photodynamic Therapy Agents. *Chemistry* **24**, 4607–4619 (2018).

[5] Dues, D. J. *et al.* Uncoupling of oxidative stress resistance and lifespan in long-lived isp-1 mitochondrial mutants in Caenorhabditis elegans. *Free Radical Biology and Medicine* **108**, 362–373 (2017).

[6] Pedersen, J. S.. C. elegans motility analysis in ImageJ ‐ A practical approach.

https://www.phage.dk/plugins/download/wrMTrck.pdf (2017).
